# The immune infiltration in clear cell Renal Cell Carcinoma and their clinical implications: A study based on TCGA and GEO databases

**DOI:** 10.7150/jca.37285

**Published:** 2020-03-05

**Authors:** Qiufeng Pan, Longwang Wang, Shuaishuai Chai, Hao Zhang, Bing Li

**Affiliations:** 1 Department of Urology, Union Hospital, Tongji Medical College, Huazhong University of Science and Technology, Wuhan 430022, China.; 2 Department of Urology, The First Affiliated Hospital of Nanchang University, Nanchang, China.

**Keywords:** devolution algorithm, tumor microenvionment, clear cell renal cell carcinoma (ccRCC), genomic signature

## Abstract

The tumor immune microenvironment in clear cell Renal Cell Carcinoma (ccRCC) still remains poorly understood. Previous methods to study the tumor immune microenvironment have a limitation when accounting for the functionally distinct cell types. In this study, we investigated the differently infiltrated immune cells and their clinical significance in ccRCC for the purpose of shedding some important light on the complex immune microenvironment in ccRCC. The devolution algorithm (CIBERSORT) was applied to infer the proportion of 22 immune infiltrating cells based on gene expression profiles of ccRCC bulk tissue, which were downloaded from TCGA and GEO databases. As a result, we observed considerable differences in immune cells percentage between ccRCC tumor tissue and paired normal tissue; meanwhile, we uncovered their internal correlations and associations with Fuhrman grade. Moreover, dendritic cells resting, dendritic cells activated, mast cells resting, mast cells activated and eosinophils were associated with favorable prognosis, whereas B cells memory, T cells follicular helper and T cells regulatory (Tregs) were correlated with poorer outcome.

## Introduction

Clear cell Renal Cell Carcinoma (ccRCC) is the most common histology identified in renal carcinoma. Its manifestations are different both biologically and in clinic 1,2. The cancer genome changes based on large scale sequencing researches contributed greatly to our understanding of underlying molecular mechanism of ccRCC 3,4. Notably, a solid tumor is not only composed of cancer cells, but also by non-cancer cells, which can profoundly influence tumor progression in an elaborate and dynamic manner 5. Among these non-cancer cells, the tumor infiltrating immune cells (TIICs) exert a central role in pro- and anti-tumorigenic processes; moreover, they have been found closely correlated with the clinical outcome and response to immunotherapy 6. Compared to other carcinomas, the roles of immune cells recruited to microenvironment in ccRCC have not yet been elucidated and still remain an enigma, especially regarding the contradictory correlation of high CD8+ T cell infiltration with poor prognosis 7,8.

Previous traditional research techniques to evaluate TIICs include immunohistochemistry and flow cytometry, both of which inevitably are limited to a narrow viewpoint when analyzing the composition of immune cells comprehensively. Additionally, with the demanding sample processing, flow cytometry may result in cytolysis of certain cell types 9,10. However, the immune response in a tumor involves in plenty of specialized cell types 11. To better understand the diversity and nature of infiltrating immune cells in ccRCC, it is a pre-requisite to enumerate the number of immune cells in an aggregative manner. CIBERSORT, a versatile gene expression-based devolution algorithm, can quantify cell fractions from gene expression profiles of bulk tissue 12. Therefore, the different types of infiltrating immune cells can be quantified simultaneously, allowing this method to obviate the concern of various surface markers and possible cellular dissociation. Due to its superiority, we used CIBERSORT in this study to enumerate 22 distinct functional immune cell types in ccRCC to define the landscape of ccRCC tumor tissue and paired normal tissue; more importantly, we investigated its relationship with other immune cells, survival and pathological grade. We hoped this study will provide some important information regarding the complex immune microenvironment in ccRCC and help to reveal new therapeutic targets.

## Materials and methods

### Data collection

**Figure 1:**
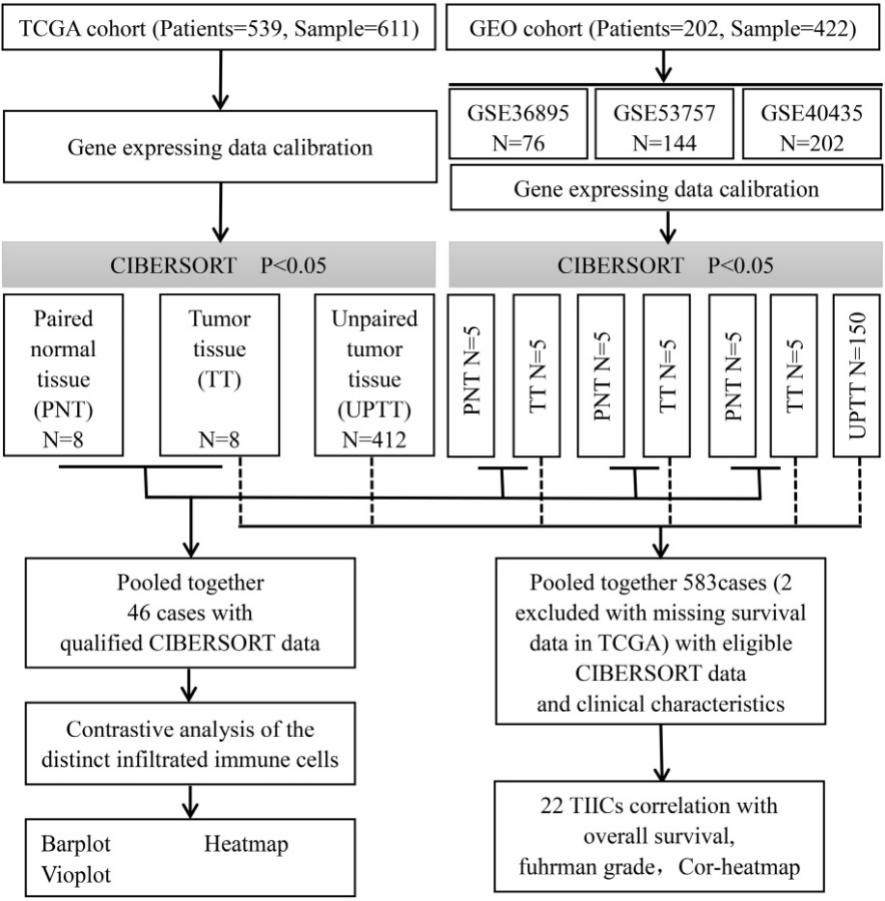
Flowchart detailing the overall study design and samples at each stage of analysis.

This study made use of data from public datasets. Gene expression profiles and corresponding clinical information from primary ccRCC tumors, uploaded up to the 31st December 2018, were downloaded from Gene Expression Omnibus (GEO) and The Cancer Genome Atlas (TCGA) 13,14. Duplicates and datasets with small sample sizes (N < 50) in GEO database were excluded. For TCGA datasets, preprocessing and aggregation of raw data were achieved by means of a robust multi-array average algorithm. Furthermore, voom (variance modelling at the observational level) was used to transform RNA sequencing data to values that are more similar to those from microarrays 15. Furthermore, gene probe names must be transformed into gene names based on platform annotation flies for GEO datasets. Subsequently, we organized each sample and corresponding clinical data for further analysis; moreover, we manually identified and picked out tumor tissue and paired normal tissue to screen differentially infiltrated immune cells to investigate whether there is a difference in the infiltrated immune cells between different tissues. The overall study design and the different samples that were included at every stage of the analysis are illustrated as a flowchart in Figure 1.

### Enumeration of tumor infiltrating immune cells

CIBERSORT is a robust analytic tool that uses gene expression signatures consisting of 547 genes. It characterizes each immune cell subtype and accurately quantifies distinct immune cell compositions using a deconvolution algorithm. The derived P-value reflects the statistical significance of deconvolution results and can help filter out samples with less significant accuracy.

Before running CIBERSORT, the original gene expression data downloaded from TCGA and GEO must be normalized as Binbin Chen et al. described previously 16. Then, the data was uploaded to the CIBERSORT web portal (http://cibersort.stanford.edu) with a number of permutations being set to 100. Relative proportions of 22 infiltrating immune cells together with CIBERSORT metrics of CIBERSORT P-value, Pearson correlation coefficient and root mean squared error (RMSE) were evaluated for each sample simultaneously.

### Statistical analysis

Only samples with a CIBERSORT P-value < 0.05 were regarded as statistically significant and included in further analysis. Correlations between different immune cell subtypes were established using the Pearson correlation coefficient. Associations between categorical and continuous variables were tested using the Kruskal-Wallis or Wilcoxon test. Survival analysis of specific immune cell subtype was conducted according to the median of the proportion of immune cell. Log-rank Mantel-Cox regression was applied to compare the survival curves between groups of patients using the Graphpad Prism 7.0 software. Additionally, multivariable analysis was adjusted according to age, gender, histological grade, T stage, lymph node metastasis, distant metastasis, and TNM stage using SPSS 24.0.

All analyses were conducted by R version 3.5.2 and all statistical tests performed were two-sided. A P-value < 0.05 was considered as statistically significant.

## Results

### The landscape of immune infiltration in ccRCC

**Figure 2:**
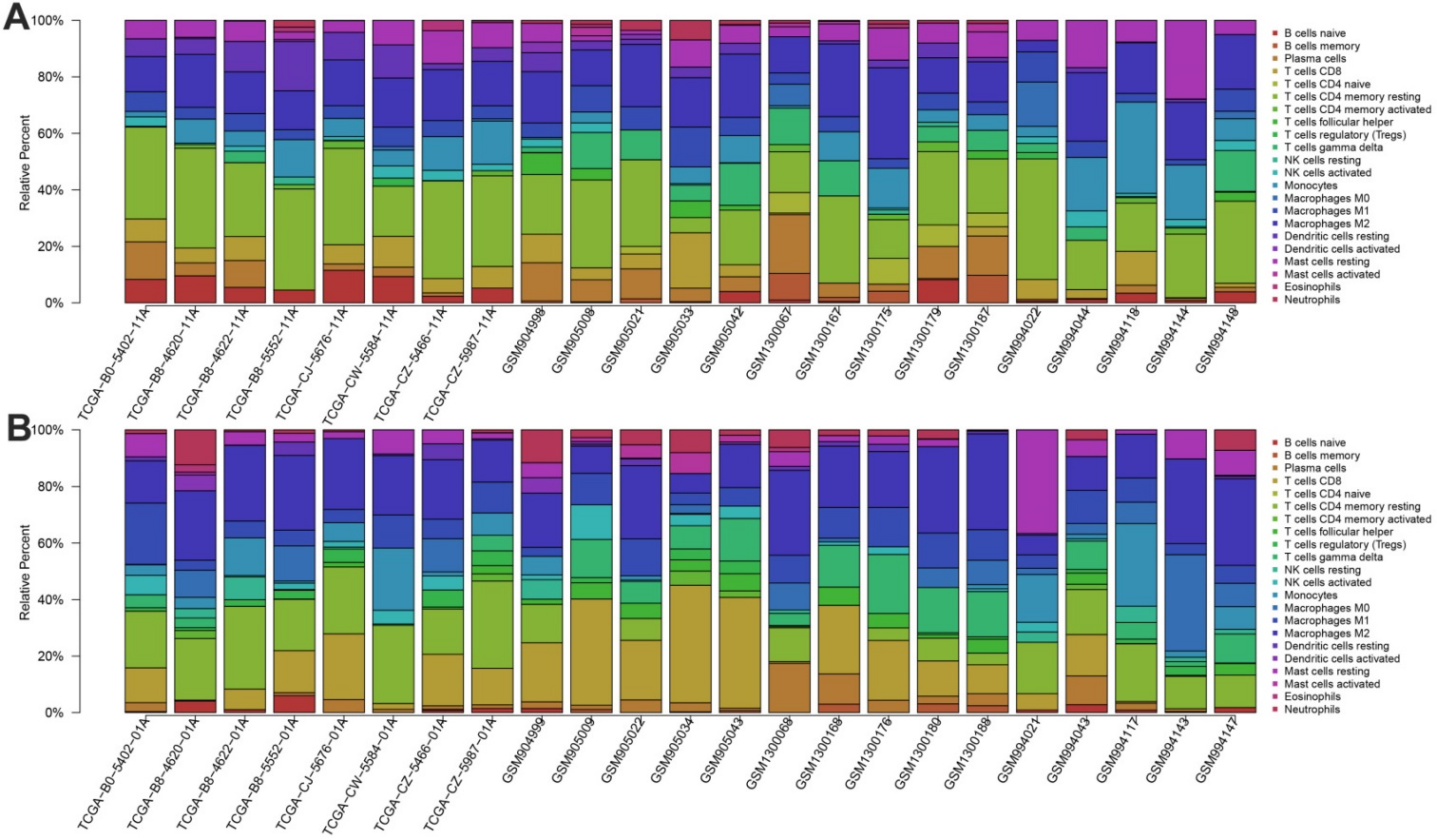
The landscape of immune infiltration in ccRCC and difference of immune infiltration between paired normal tissue and tumor tissue in ccRCC. **A.** Paired normal tissue; **B.** Tumor tissue.

**Figure 3:**
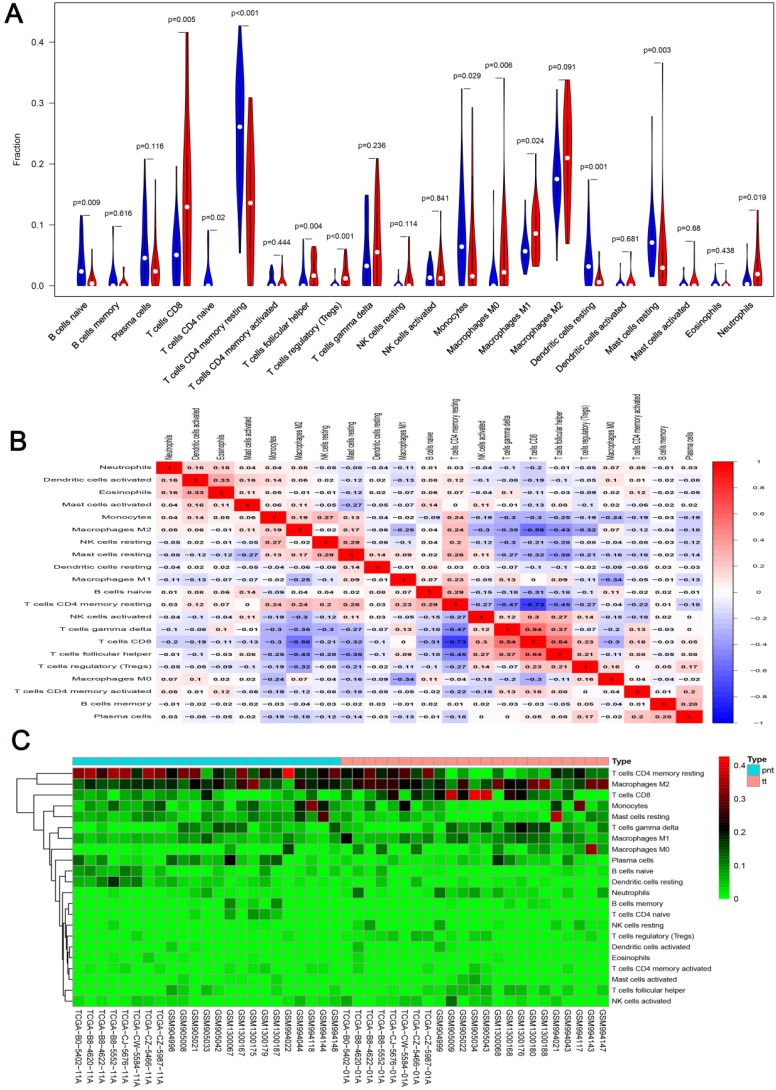
** A.** Violin plot visualizing the differentially infiltrated immune cells; **B.** Correlation heatmap depicting correlations between infiltrated immune cells in tumor; **C.** Heat map of the 22 immune cell proportions.

We first revealed the landscape of 22 immune cell subpopulations infiltration in ccRCC, and subsequently we investigated the difference between tumor tissue and paired normal tissue using the CIBERSORT algorithm. Detailed results are presented in Table 1. The fraction of immune cells varied distinctly between groups (Figure 2A, 2B). Compared with paired normal tumor tissue, ccRCC tissue contained a greater number of T cells CD8+, T cells follicular helper, T cells regulator (Tregs), Macrophages M0, Macrophages M1 and neutrophils. However, B cells naive, T cells CD4 naive, T cells CD4 memory resting, monocytes, dendritic cells resting and mast cells resting fractions were relative lower (Figure 3A). The proportions of 22 TIICs were weakly-to-strongly correlated in tumor. T cells CD8+ and T cells follicular helper showed the strongest positive correlation (Pearson correlation = 0.54), while T cells CD8+ and T cells CD4+ memory resting showed the strongest negative correlation (Pearson correlation = 0.73); moreover, T cells CD8+ also indicated moderate negative correlation with Macrophages M2 (Pearson correlation = 0.56) (Figure 3B). Altogether, these results revealed that the immune response of ccRCC acted as an intricate network and proceeded in a tightly regulated way.

### Identification of clinical implications of TIICs subsets

**Figure 4:**
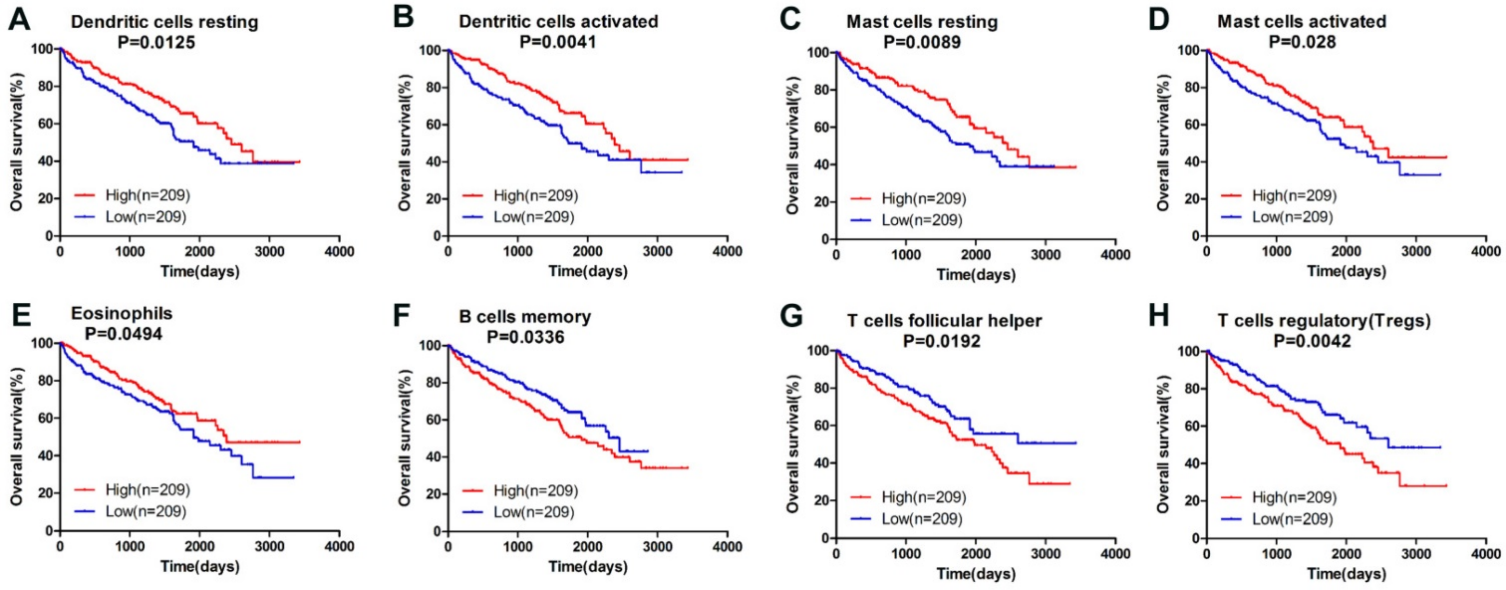
Survival plots of median of immune cell subpopulations with *P*-value < 0.05.

Owning to the missing survival data in included GEO datasets, we investigated whether there was a statistical relationship between specific TIICs and ccRCC overall survival obtained from TCGA by univariate Cox regression through Graphpad Prism7.0. After a restriction of CIBERSORT filter to *P* < 0.05, there were 418 patients with available data on overall survival (142 events). The detailed 95% confidence intervals, unadjusted HRs and *P*-value for the median fractions of TIICs subtypes are presented in Table 2. Dendritic cells resting ([HR] = 0.656; 95% CI = 0.471-0.913; *P=*0.013 ), dendritic cells activated (hazard ratio [HR] = 0.617; 95% CI = 0.443-0.858; *P=*0.004 ), mast cells resting ([HR] = 0.647; 95% CI = 0.465-0.900; *P=*0.010 ), mast cells activated ([HR] = 0.690; 95% CI = 0.496-0.091; *P=*0.028 ) and eosinophils ([HR] = 0.728; 95% CI = 0.516-0.999; *P=*0.049 ) were significantly associated with a favorable outcome. In contrast, B cells memory ([HR] = 1.437; 95% CI = 1.028-2.008; *P=*0.034 ), T cells follicular helper ([HR] = 1.485; 95% CI = 1.067-2.067; *P=*0.019 ) and T cells regulatory (Tregs) ([HR] = 1.621; 95% CI = 1.165-2.256; *P=*0.004 ) were associated with poorer outcome. The corresponding Kaplan-Meier curve and Log-rank test are depicted in Figure 4. Among those TIICs subtypes associated with overall survival, we evaluated their possibility of becoming independent prognostic factor using multivariable analysis adjusted for known prognostic factors. However, none represented an independent prognostic factor besides T stage (T1-T2/T3-T4), lymph node metastasis (N0/N1-N2), distant metastasis (M0/M1) and TNM stage.

**Figure 5:**
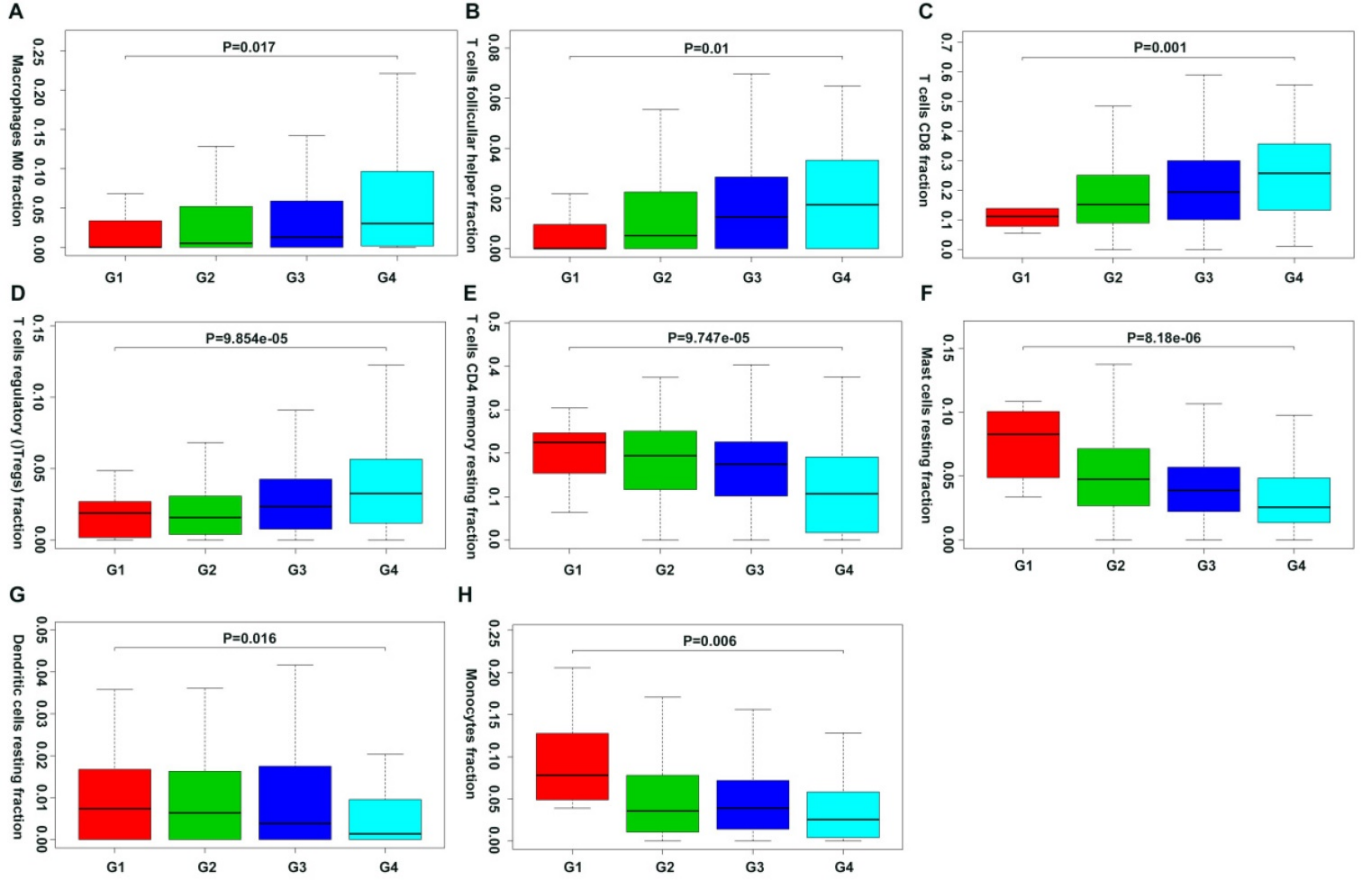
Correlation of specific immune cell proportions with Fuhrman grade in ccRCC.

Moreover, we first revealed the association between different immune cell subsets and ccRCC pathological grade by combing the clinical characteristics of TCGA and GEO databases. Result showed that the fraction of dendritic cells resting, mast cells resting, monocytes, T cells CD4+ memory resting decreased with the increasing Fuhrman grade, whereas the fraction of T cells CD8+, T cells follicular helper, T cells regulatory (Tregs) and Macrophages M0 increased with the elevated Fuhrman grade (Figure 5; Supplementary Table 1).

## Discussion

The tumor microenvironment, which comprises malignant tumor cells, various infiltrating immune cells, fibroblasts and numerous cytokines and chemokines, is well recognized as a complex biological process like an intricate and dynamic ecosystem. Among this ecosystem, immune response plays an important role in tumor growth, invasion and metastasis; therefore, it is treated as another therapeutic target beyond chemotherapy and radiation 11. However, despite the astounding clinical successes in multiple tumor types, many more patients experienced minimal or no clinical response to the same immunotherapeutic intervention 17. Until recently, the roles of TIICs have not been fully understood. Hence it is of vital importance to figure out the diversity and complexity of the tumor immune context to predict and guide immunotherapy, as well as to reveal novel biomarkers and targets for therapeutic modulation.

With the advances in computational methods, a deconvolution algorithm called CIBERSORT was developed, which could infer the proportions of 22 TIICs subpopulations from tumor transcriptomes. This method was validated by FACS successfully and was conducted in breast cancer, and lung cancer patients 18,19. In this study, we applied CIBERSORT to uncover distinct patterns of TIICs in ccRCC and associations of different immune cells subsets with clinical outcomes.

We observed significant differences in immune cell composition between ccRCC tumor tissue and paired normal tissue. Our data revealed the detailed profile of 22 TIICs subtypes infiltration in ccRCC that the proportions of total T cells accounted for more than 40%, in which the CD8+ T cells comprised of 14.8%. Secondly, the proportions of total macrophages accounted for more than 30%, in which 20.9% were M2 cells. Moreover, our work confirmed the findings that certain immune cells subsets can also predict clinical outcomes beyond the immunoscore. By univariate Cox regression analysis, we found that dendritic cells resting, dendritic cells activated, mast cells resting, mast cells activated and eosinophils are significantly associated with improved outcome, while B cells memory, T cells follicular helper and T cells regulatory (Tregs) indicate poorer outcome.

It is commonly believed that CD8+ T cells can recognize tumor specific antigens and play a role in tumor control 20. High densities of tumor infiltrated CD8+ T cells are shown to be associated with favorable prognosis in vast majority of cancers 5; therefore, many forms of immunotherapy aim at restoring T-cell mediated immune response 17. Previous studies described ccRCC as a pro-inflammatory tumor where malignant cells and infiltrated neutrophils produce various kinds of cytokines that may help recruit and activate polyclonal CD8+ T cells 21-23. Paradoxically, CD8+ T cells infiltration in ccRCC correlates with a poorer prognosis. Nicolas A. Giraldo et al. revealed that recruited CD8+ T cells correlated with favorable prognosis only with the present of fully functional mature dendritic cells (DC) 7. Moreover, several studies indicated that dysfunctional DC maturation can be induced in the ccRCC microenvironment by down-regulating co-stimulatory molecules 24-26. Recently, Kondou R et al. discovered that in PD-L1+ and CD8B+ patients, the gene expression profiling of fresh specimens exhibited an upregulation of dendritic cell maturation genes and T-cell activation genes. However, patients with PD-L1- and CD8B- exhibited a low expression of T-cell-activation genes 27. Meanwhile, our results demonstrated that dendritic resting cells and dendritic activated cells are associated with favorable prognosis. These results revealed that malfunction of DC might involve in the process of T cells inhibition and become a potential combined therapeutic target.

Furthermore, research in human lung and colorectal cancer showed that CD8+ T cells are not only specific for tumor-derived antigens; but also recognize a variety of epitopes unrelated to cancer 28. They demonstrated that abundant CD8+ T cells act as bystanders and are phenotypically heterogeneous within a tumor and across patients. From this perspective, the role of CD8+ T cells level in predicting prognosis differs between patients. Collectively, these explain the possible reasons for the correlation of CD8+ T cells infiltration with poor outcome. In our study, however, we did not find association between CD8+ T cells and overall survival; besides, the percentages of neutrophils in the entire TIICs was uncorrelated with clinical outcome, but Jensen HK et al. revealed that the presence of intramural neutrophils correlates with poor prognosis 29. The discrepancy may be ascribed to the nature of algorithm, which lack information related to cellular heterogeneity and deeper spatial distribution.

Previous studies regarding regulatory T cells and Macrophages M2 showed that they all exert pro-tumorigenic function 30,31. Our study revealed that T cells regulatory and T cells follicular helper are associated with poor prognosis. Interestingly, we also found the positive relationship between specific immune cells proportion and ccRCC Fuhrman grade including CD8+ T cells, T cells regulatory and T cells follicular helper. Moreover, corHeatmap indicated negative correlation between CD8+ T cells and Macrophages M2. Collectively, our work indicated that Macrophages M2, T cells regulatory and T cells follicular helper possibly play a role in T cell exhaustion/inhibition in an intricated “checks and balances” mannner 32.

Mast cells and eosinophils have been studied mainly in the allergic disease. It has been reported that mast cells and eosinophils can exhibit both tumor promoting and anti-tumor activity 33,34. Underlying molecular mechanisms of the contradictory function have remained a conundrum. Increased mast cell infiltration predicts a poorer prognosis in many cancers including lung, colorectal, gastric, melanoma and cervical carcinoma 35; nevertheless, mast cell infiltration is associated with improved prognosis for breast carcinoma and prostate cancer 36,37. Our study revealed that infiltration of resting mast cells, activated mast cells and eosinophils are correlated with favorable prognosis in ccRCC. However, the fraction of resting mast cells decreased with the increased Fuhrman grade. With the merits of a low rate of cell division and long lifespan, mast cells are eligible alternative candidates for combined targeted immunotherapy. Moreover, Hollande et al. first demonstrated that using T cell- and eosinophils-targeted combination therapy yields increased tumor retardation, which advances our understanding of the anti-tumor role of eosinophils 38.

Nevertheless, our study has several limitations. We pooled together data from TCGA and GEO to enlarge our sample size, which may affect the repeatability of results on account of the heterogeneity. Secondly, our results are based on public database and computational algorithm. Although the accuracy of this technique has been testified using FACS, it is still required to further verify them by experiments in the future.

In conclusion, our analysis, based on a devolution algorithm, revealed significant differences in the cellular composition of infiltrated immune cells in ccRCC and associations between 22 immune cells subpopulations and clinical outcome. Particularly, DC, mast cells, eosinophils, T cells regulatory and T cells follicular helper emerge as potential immunotherapy targets. Moreover, the CIBERSORT algorithm makes the comprehensive analysis of ccRCC immune microenvironment possible by using gene expression data from bulk tissues.

## Supplementary Material

Supplementary table.

## Figures and Tables

**Table 1:** Comparison of 22 TIICs proportion between ccRCC and paired normal tissue

Cell type	CIBERSORT fraction in % of all infiltrating immune cells (mean± SD)		
	Paired normal tissue	Tumor tissue	
B cells naive	0.035 ± 0.037	0.009 ± 0.015	**0.009**
B cells memory	0.012 ± 0.028	0.005± 0.010	0.616
T cells CD8	0.054 ± 0.048	0.148 ± 0.126	**0.005**
T cells CD4 naive	0.014 ± 0.029	0.000 ± 0.000	**0.020**
T cells CD4 memory resting	0.256 ± 0.091	0.137 ± 0.096	**<0.001**
T cells CD4 memory activated	0.008 ± 0.011	0.006 ± 0.013	0.444
T cells follicular helper	0.011 ± 0.022	0.024 ± 0.024	**0.004**
T cells regulatory (Tregs)	0.002 ± 0.007	0.018 ± 0.019	**<0.001**
T cells gamma delta	0.048 ± 0.055	0.069 ± 0.061	0.236
NK cells resting	0.003 ± 0.007	0.014 ± 0.024	0.114
NK cells activated	0.017 ± 0.017	0.023 ± 0.003	0.841
Monocytes	0.089 ± 0.075	0.056 ± 0.078	**0.029**
Macrophages M0	0.012 ± 0.036	0.052 ± 0.077	**0.006**
Macrophages M1	0.060 ± 0.027	0.088 ± 0.044	**0.024**
Macrophages M2	0.175 ± 0.056	0.209 ± 0.079	0.091
Dendritic cells resting	0.044 ± 0.044	0.010 ± 0.015	**0.001**
Dendritic cells activated	0.003 ± 0.008	0.006 ± 0.016	0.681
Mast cells resting	0.080 ± 0.055	0.051 ± 0.076	**0.003**
Mast cells activated	0.004 ± 0.009	0.007 ± 0.018	0.680
Plasma cells	0.059 ± 0.057	0.034 ± 0.042	0.116
Eosinophils	0.004 ± 0.009	0.002 ± 0.006	0.438
Neutrophils	0.009 ± 0.016	0.031 ± 0.037	**0.019**

Significance of bold values are p < 0.05.

**Table 2:** Prognostic associations of 22 TIICs subpopulation

Tumor infiltrating immune cells	Hazard Ratio	95% CI of ratio	P-value
T cells CD4 naive	—	—	—
Dendritic cells activated	0.617	[ 0.443 ; 0.858 ]	0.004
Mast cells resting	0.647	[ 0.465 ; 0.900 ]	0.010
Dendritic cells resting	0.656	[ 0.471 ; 0.913 ]	0.013
Mast cells activated	0.690	[ 0.496 ; 0.091 ]	0.028
Monocytes	0.725	[ 0.521 ; 1.008 ]	0.056
Eosinophils	0.728	[ 0.516 ; 0.999 ]	0.049
Macrophages M2	0.765	[ 0.550 ; 1.064 ]	0.112
T cells CD4 memory resting	0.888	[ 0.638 ; 1.236 ]	0.481
Neutrophils	0.917	[ 0.659 ; 1.276 ]	0.607
NK cells activated	0.934	[ 0.673 ; 1.303 ]	0.698
T cells CD8	0.973	[ 0.699 ; 1.354 ]	0.870
T cells gamma delta	0.983	[ 0.706 ; 1.369 ]	0.918
B cells naive	1.065	[ 0.765 ; 1.482 ]	0.708
Plasma cells	1.170	[ 0.841 ; 1.628 ]	0.352
NK cells resting	1.186	[ 0.851 ; 1.655 ]	0.314
Macrophages M0	1.198	[ 0.860 ; 1.667 ]	0.085
Macrophages M1	1.226	[ 0.881 ; 1.707 ]	0.226
T cells CD4 memory activated	1.337	[ 0.960 ; 1.862 ]	0.086
B cells memory	1.437	[ 1.028 ; 2.008 ]	0.034
T cells follicular helper	1.485	[ 1.067 ; 2.067 ]	0.019
T cells regulatory (Tregs)	1.621	[ 1.165 ; 2.256 ]	0.004

Significance of bold values are p < 0.05.

## References

[1] Ljungberg B, Albiges L, Abu-Ghanem Y (2019). European Association of Urology Guidelines on Renal Cell Carcinoma: The 2019 Update.

[2] Siegel RL, Miller KD, Jemal A (2017). Cancer Statistics, 2017. CA Cancer J Clin.

[3] Dahinden C, Ingold B, Wild P (2010). Mining tissue microarray data to uncover combinations of biomarker expression patterns that improve intermediate staging and grading of clear cell renal cell cancer. Clin Cancer Res.

[4] Gerlinger M, Horswell S, Larkin J (2014). Genomic architecture and evolution of clear cell renal cell carcinomas defined by multiregion sequencing. Nat Genet.

[5] Fridman WH, Pages F, Sautes-Fridman C, Galon J (2012). The immune contexture in human tumours: impact on clinical outcome. Nat Rev Cancer.

[6] Chen DS, Mellman I (2017). Elements of cancer immunity and the cancer-immune set point. Nature.

[7] Giraldo NA, Becht E, Pages F (2015). Orchestration and Prognostic Significance of Immune Checkpoints in the Microenvironment of Primary and Metastatic Renal Cell Cancer. Clin Cancer Res.

[8] Nakano O, Sato M, Naito Y (2001). Proliferative activity of intratumoral CD8(+) T-lymphocytes as a prognostic factor in human renal cell carcinoma: clinicopathologic demonstration of antitumor immunity. Cancer Res.

[9] Adan A, Alizada G, Kiraz Y, Baran Y, Nalbant A (2017). Flow cytometry: basic principles and applications. Crit Rev Biotechnol.

[10] Mori H, Cardiff RD (2016). Methods of Immunohistochemistry and Immunofluorescence: Converting Invisible to Visible. Methods Mol Biol.

[11] Binnewies M, Roberts EW, Kersten K (2018). Understanding the tumor immune microenvironment (TIME) for effective therapy. Nat Med.

[12] Newman AM, Liu CL, Green MR (2015). Robust enumeration of cell subsets from tissue expression profiles. Nat Methods.

[13] Edgar R, Domrachev M, Lash AE (2002). Gene Expression Omnibus: NCBI gene expression and hybridization array data repository. Nucleic Acids Res.

[14] Wei L, Jin Z, Yang S, Xu Y, Zhu Y, Ji Y (2018). TCGA-assembler 2: software pipeline for retrieval and processing of TCGA/CPTAC data. Bioinformatics.

[15] Ritchie ME, Phipson B, Wu D (2015). limma powers differential expression analyses for RNA-sequencing and microarray studies. Nucleic Acids Res.

[16] Chen B, Khodadoust MS, Liu CL, Newman AM, Alizadeh AA (2018). Profiling Tumor Infiltrating Immune Cells with CIBERSORT. Methods Mol Biol.

[17] Yang Y (2015). Cancer immunotherapy: harnessing the immune system to battle cancer. J Clin Invest.

[18] Ali HR, Chlon L, Pharoah PD, Markowetz F, Caldas C (2016). Patterns of Immune Infiltration in Breast Cancer and Their Clinical Implications: A Gene-Expression-Based Retrospective Study. PLoS Med.

[19] Liu X, Wu S, Yang Y, Zhao M, Zhu G, Hou Z (2017). The prognostic landscape of tumor-infiltrating immune cell and immunomodulators in lung cancer. Biomed Pharmacother.

[20] Chen DS, Mellman I (2013). Oncology meets immunology: the cancer-immunity cycle. Immunity.

[21] Rosales C (2018). Neutrophil: A Cell with Many Roles in Inflammation or Several Cell Types?. Front Physiol.

[22] Shabtai M, Ye H, Frischer Z, Martin J, Waltzer WC, Malinowski K (2002). Increased expression of activation markers in renal cell carcinoma infiltrating lymphocytes. J Urol.

[23] Sittig SP, Kollgaard T, Gronbaek K (2013). Clonal expansion of renal cell carcinoma-infiltrating T lymphocytes. Oncoimmunology.

[24] Gigante M, Blasi A, Loverre A (2009). Dysfunctional DC subsets in RCC patients: ex vivo correction to yield an effective anti-cancer vaccine. Mol Immunol.

[25] Teng L, Chen Y, Ding D, Dai H, Liu G, Li C (2014). Immunosuppressive effect of renal cell carcinoma on phenotype and function of dendritic cells. Int Urol Nephrol.

[26] Troy AJ, Summers KL, Davidson PJ, Atkinson CH, Hart DN (1998). Minimal recruitment and activation of dendritic cells within renal cell carcinoma. Clin Cancer Res.

[27] Kondou R, Iizuka A, Nonomura C (2019). Classification of tumor microenvironment immune types based on immune response-associated gene expression. Int J Oncol.

[28] Simoni Y, Becht E, Fehlings M (2018). Bystander CD8(+) T cells are abundant and phenotypically distinct in human tumour infiltrates. Nature.

[29] Jensen HK, Donskov F, Marcussen N, Nordsmark M, Lundbeck F, von der Maase H (2009). Presence of intratumoral neutrophils is an independent prognostic factor in localized renal cell carcinoma. J Clin Oncol.

[30] Finotello F, Trajanoski Z (2017). New strategies for cancer immunotherapy: targeting regulatory T cells. Genome Med.

[31] She L, Qin Y, Wang J (2018). Tumor-associated macrophages derived CCL18 promotes metastasis in squamous cell carcinoma of the head and neck. Cancer Cell Int.

[32] Speiser DE, Utzschneider DT, Oberle SG, Munz C, Romero P, Zehn D (2014). T cell differentiation in chronic infection and cancer: functional adaptation or exhaustion?. Nat Rev Immunol.

[33] Oldford SA, Marshall JS (2015). Mast cells as targets for immunotherapy of solid tumors. Mol Immunol.

[34] Reichman H, Karo-Atar D, Munitz A (2016). Emerging Roles for Eosinophils in the Tumor Microenvironment. Trends Cancer.

[35] Groot Kormelink T, Abudukelimu A, Redegeld FA (2009). Mast cells as target in cancer therapy. Curr Pharm Des.

[36] Fleischmann A, Schlomm T, Kollermann J (2009). Immunological microenvironment in prostate cancer: high mast cell densities are associated with favorable tumor characteristics and good prognosis. Prostate.

[37] Rajput AB, Turbin DA, Cheang MC (2008). Stromal mast cells in invasive breast cancer are a marker of favourable prognosis: a study of 4,444 cases. Breast Cancer Res Treat.

[38] Hollande C, Boussier J, Ziai J (2019). Inhibition of the dipeptidyl peptidase DPP4 (CD26) reveals IL-33-dependent eosinophil-mediated control of tumor growth. Nat Immunol.

