# Supplementary material for: The immune infiltration in clear cell Renal Cell Carcinoma and their clinical implications: A study based on TCGA and GEO databases
Source: J Cancer. 2020 Mar 5;11(11):3207–15. doi: 10.7150/jca.37285 (PMC7097965; doi:10.7150/jca.37285)
Supplement: Supplementary file 1 — Supplementary table. [file jcav11p3207s1.pdf]

Supplementary table 1. Corresponding information of 583 enrolled patients

ID	Time(days)	State	age	gender	grade	stage	T	M	N
**TCGA-6D-AA2E-01A**	362	0	68	FEMALE	G2	Stage I	T1b	MX	NX
**TCGA-A3-3306-01A**	1120	0	67	MALE	G3	Stage I	T1b	M0	N0
**TCGA-A3-3307-01A**	1436	0	66	MALE	G3	Stage III	T3b	M0	N0
**TCGA-A3-3308-01A**	16	0	77	FEMALE	G2	Stage III	T3b	M0	N0
**TCGA-A3-3316-01A**	1493	0	57	MALE	G3	Stage II	T2	M0	NX
**TCGA-A3-3317-01A**	1491	0	67	MALE	G2	Stage II	T2	M0	N0
**TCGA-A3-3319-01A**	1130	0	70	MALE	G2	Stage I	T1b	M0	NX
**TCGA-A3-3322-01A**	1478	0	51	MALE	G2	Stage I	T1a	M0	NX
**TCGA-A3-3323-01A**	1106	0	53	MALE	G1	Stage I	T1b	M0	NX
**TCGA-A3-3324-01A**	1186	0	51	MALE	G3	Stage I	T1b	M0	NX
**TCGA-A3-3325-01A**	1170	1	52	MALE	G2	Stage I	T1a	M0	NX

**TCGA-A3-3326-01A**	1137	0	47	MALE	G1	Stage I	T1a	M0	NX
**TCGA-A3-3329-01A**	1624	0	75	MALE	G2	Stage I	T1b	M0	N0
**TCGA-A3-3331-01A**	1257	0	86	FEMALE	G2	Stage I	T1	M0	N0
**TCGA-A3-3343-01A**	945	0	79	MALE	G3	Stage II	T2	M0	N0
**TCGA-A3-3346-01A**	137	1	68	MALE	G3	Stage I	T1b	M0	NX
**TCGA-A3-3347-01A**	1610	1	76	FEMALE	G2	Stage III	T1b	M0	N1
**TCGA-A3-3349-01A**	1385	0	34	FEMALE	G2	Stage I	T1b	M0	N0
**TCGA-A3-3351-01A**	910	0	42	MALE	G2	Stage II	T2a	M0	N0
**TCGA-A3-3357-01A**	2688	0	62	MALE	G3	Stage II	T2	M0	N0
**TCGA-A3-3358-01A**	1307	0	57	FEMALE	G2	Stage I	T1a	M0	N0
**TCGA-A3-3359-01A**	2504	0	82	FEMALE	G2	Stage I	T1a	M0	N0
**TCGA-A3-3362-01A**	1559	0	60	FEMALE	G2	Stage I	T1a	M0	N0
**TCGA-A3-3363-01A**	319	0	50	MALE	G2	Stage II	T2	M0	N0

**TCGA-A3-3365-01A**	873	0	46	MALE	G2	Stage I	T1a	M0	NX
**TCGA-A3-3370-01A**	2274	0	48	FEMALE	G2	Stage I	T1b	M0	N0
**TCGA-A3-3372-01A**	735	0	64	MALE	G2	Stage III	T3	M0	NX
**TCGA-A3-3373-01A**	1621	0	54	FEMALE	G3	Stage I	T1b	M0	N0
**TCGA-A3-3374-01A**	1314	0	51	FEMALE	G2	Stage I	T1b	M0	N0
**TCGA-A3-3376-01A**	1696	1	51	MALE	G2	Stage I	T1a	M0	N0
**TCGA-A3-3378-01A**	630	0	60	MALE	G3	Stage I	T1	M0	N0
**TCGA-A3-3380-01A**	567	0	54	MALE	G2	Stage I	T1	M0	N0
**TCGA-A3-3382-01A**	574	0	69	MALE	G3	Stage I	T1b	M0	NX
**TCGA-A3-3387-01A**	617	0	49	MALE	G2	Stage I	T1a	M0	N0
**TCGA-A3-A6NJ-01A**	468	0	57	FEMALE	G1	Stage I	T1a	MX	NX
**TCGA-A3-A6NL-01A**	689	0	49	FEMALE	G2	Stage I	T1b	MX	NX
**TCGA-A3-A6NN-01A**	3	0	78	MALE	G2	Stage I	T1a	MX	NX

**TCGA-A3-A8OU-01A**	0	0	74	FEMALE	G1	Stage I	T1a	MX	NX
**TCGA-A3-A8OW-01A**	323	0	37	MALE	G2	Stage III	T3a	MX	NX
**TCGA-A3-A8OX-01A**	0	0	65	FEMALE	G3	Stage I	T1a	MX	NX
**TCGA-AK-3425-01A**	3343	0	68	MALE	G2	Stage I	T1	M0	N0
**TCGA-AK-3426-01A**	885	1	37	MALE	G3	Stage III	T3a	M0	N1
**TCGA-AK-3428-01A**	2223	0	62	MALE	G2	Stage III	T3b	M0	N0
**TCGA-AK-3429-01A**	2017	0	54	FEMALE	G2	Stage II	T2	M0	N0
**TCGA-AK-3431-01A**	1853	0	62	FEMALE	G3	Stage II	T2	M0	NX
**TCGA-AK-3434-01A**	2087	0	72	MALE	G2	Stage I	T1b	M0	NX
**TCGA-AK-3436-01A**	2044	0	40	MALE	G2	Stage IV	T2	M1	N0
**TCGA-AK-3445-01A**	1280	0	69	MALE	G3	Stage III	T3a	M0	NX
**TCGA-AK-3451-01A**	1481	0	48	MALE	G3	Stage II	T2	M0	N0
**TCGA-AK-3453-01A**	1397	0	58	FEMALE	G2	Stage II	T2	M0	NX

**TCGA-AK-3454-01A**	874	0	84	MALE	G3	Stage I	T1b	M0	NX
**TCGA-AK-3455-01A**	683	1	71	FEMALE	G3	Stage III	T3b	M0	NX
**TCGA-AK-3456-01A**	1143	0	48	MALE	G3	Stage II	T2	M0	N0
**TCGA-AK-3458-01A**	1168	0	48	MALE	G3	Stage I	T1b	M0	NX
**TCGA-AK-3460-01A**	951	0	58	MALE	G2	Stage I	T1a	M0	NX
**TCGA-AS-3778-01A**	43	0	35	MALE	G1	Stage I	T1a	M0	NX
**TCGA-B0-4690-01A**	43	1	65	MALE	G3	Stage IV	T4	M1	N0
**TCGA-B0-4691-01A**	139	1	55	MALE	G3	Stage IV	T2	M1	N0
**TCGA-B0-4693-01A**	77	1	72	FEMALE	G4	Stage III	T3a	M0	N0
**TCGA-B0-4694-01A**	106	1	72	MALE	G4	Stage III	T3b	M0	NX
**TCGA-B0-4697-01A**	578	1	46	FEMALE	G4	Stage IV	T3b	M1	NX
**TCGA-B0-4698-01A**	42	1	75	MALE	G4	Stage IV	T4	M0	NX
**TCGA-B0-4699-01A**	110	1	74	MALE	G4	Stage IV	T4	M1	N0

**TCGA-B0-4700-01A**	1980	1	60	MALE	G4	Stage IV	T4	M1	NX
**TCGA-B0-4701-01A**	238	1	66	FEMALE	G3	Stage IV	T3a	M1	N0
**TCGA-B0-4703-01A**	182	1	51	MALE	G4	Stage IV	T3a	M1	N0
**TCGA-B0-4706-01A**	65	1	61	MALE	G4	Stage III	T3a	M0	NX
**TCGA-B0-4707-01A**	600	1	63	MALE	G4	Stage III	T3a	M0	NX
**TCGA-B0-4710-01A**	96	0	75	FEMALE	G3	Stage III	T3a	M0	N0
**TCGA-B0-4712-01A**	1337	1	76	MALE	G3	Stage IV	T3a	M1	NX
**TCGA-B0-4713-01A**	202	1	76	FEMALE	G2	Stage III	T3b	M0	NX
**TCGA-B0-4714-01A**	99	1	81	MALE	G3	Stage IV	T3b	M1	NX
**TCGA-B0-4718-01A**	616	0	57	MALE	G2	Stage III	T3a	M0	NX
**TCGA-B0-4810-01A**	478	1	47	MALE	G3	Stage III	T3a	M0	N1
**TCGA-B0-4811-01A**	1417	1	48	MALE	G3	Stage III	T3a	M0	N0
**TCGA-B0-4813-01A**	18	1	68	MALE	G3	Stage III	T3b	M0	NX

**TCGA-B0-4815-01A**	1588	1	65	MALE	G4	Stage III	T3a	M0	NX
**TCGA-B0-4816-01A**	1371	1	49	MALE	G3	Stage II	T2	M0	N0
**TCGA-B0-4817-01A**	1019	1	81	MALE	G3	Stage III	T3c	M0	N0
**TCGA-B0-4818-01A**	510	1	68	FEMALE	G3	Stage II	T2	M0	NX
**TCGA-B0-4819-01A**	183	1	60	FEMALE	G4	Stage IV	T3b	M1	NX
**TCGA-B0-4821-01A**	1230	1	68	FEMALE	G3	Stage III	T3b	M0	N0
**TCGA-B0-4822-01A**	1111	1	78	MALE	G4	Stage II	T2	M0	NX
**TCGA-B0-4823-01A**	454	1	88	MALE	G2	Stage I	T1a	M0	N0
**TCGA-B0-4824-01A**	1657	1	49	FEMALE	G3	Stage I	T1a	M0	N0
**TCGA-B0-4827-01A**	885	1	77	FEMALE	G4	Stage III	T3b	M0	N0
**TCGA-B0-4828-01A**	307	1	79	MALE	G3	Stage IV	T2	M1	NX
**TCGA-B0-4833-01A**	2386	1	82	FEMALE	G2	Stage I	T1b	M0	N0
**TCGA-B0-4836-01A**	1238	1	61	MALE	G3	Stage IV	T3b	M1	NX

**TCGA-B0-4837-01A**	1378	1	63	MALE	G3	Stage I	T1b	M0	N0
**TCGA-B0-4838-01A**	834	1	69	FEMALE	G3	Stage I	T1b	M0	N0
**TCGA-B0-4839-01A**	1639	1	80	FEMALE	G2	Stage I	T1b	M0	N0
**TCGA-B0-4841-01A**	204	1	63	MALE	G3	Stage IV	T2	M1	NX
**TCGA-B0-4842-01A**	1724	1	73	FEMALE	G4	Stage III	T3a	M0	N0
**TCGA-B0-4843-01A**	320	1	57	MALE	G3	Stage III	T3a	M0	N0
**TCGA-B0-4844-01A**	313	1	60	MALE	G3	Stage IV	T3a	M1	NX
**TCGA-B0-4846-01A**	1200	1	52	MALE	G2	Stage IV	T3a	M1	N0
**TCGA-B0-4847-01A**	793	1	60	MALE	G3	Stage IV	T3a	M1	NX
**TCGA-B0-4848-01A**	883	1	54	MALE	G3	Stage III	T3b	M0	NX
**TCGA-B0-4849-01A**	69	1	51	MALE	G3	Stage III	T3a	M0	NX
**TCGA-B0-4852-01A**	1121	1	78	FEMALE	G2	Stage II	T2	M0	N0
**TCGA-B0-4945-01A**	2145	1	75	FEMALE	G2	Stage I	T1a	M0	N0

**TCGA-B0-5075-01A**	637	1	77	FEMALE	G2	Stage III	T3a	M0	N0
**TCGA-B0-5077-01A**	1317	1	77	MALE	G3	Stage I	T1a	M0	N0
**TCGA-B0-5080-01A**	342	1	63	MALE	G3	Stage IV	T3a	M1	N0
**TCGA-B0-5081-01A**	362	1	79	FEMALE	G2	Stage III	T3b	M0	N0
**TCGA-B0-5083-01A**	1045	1	63	MALE	G3	Stage I	T1a	M0	N0
**TCGA-B0-5084-01A**	222	1	33	MALE	G3	Stage IV	T3a	M1	N1
**TCGA-B0-5088-01A**	563	1	53	MALE	G3	Stage I	T1b	M0	N0
**TCGA-B0-5092-01A**	459	1	53	FEMALE	G3	Stage IV	T1a	M1	N0
**TCGA-B0-5095-01A**	245	1	81	MALE	G3	Stage III	T3a	M0	N0
**TCGA-B0-5096-01A**	68	1	72	FEMALE	GX	Stage III	T3a	M0	N1
**TCGA-B0-5097-01A**	665	0	59	FEMALE	G2	Stage III	T3b	M0	N0
**TCGA-B0-5100-01A**	1913	1	72	MALE	G3	Stage III	T3a	M0	NX
**TCGA-B0-5102-01A**	2764	1	74	FEMALE	G3	Stage I	T1	M0	NX

**TCGA-B0-5106-01A**	1598	1	64	MALE	G2	Stage I	T1a	M0	N0
**TCGA-B0-5107-01A**	927	1	65	FEMALE	G4	Stage IV	T2	M1	N0
**TCGA-B0-5108-01A**	911	0	54	MALE	G2	Stage III	T3a	M0	N0
**TCGA-B0-5109-01A**	587	1	69	MALE	G4	Stage III	T3b	M0	N1
**TCGA-B0-5113-01A**	359	0	69	FEMALE	G2	Stage III	T3a	M0	N0
**TCGA-B0-5115-01A**	797	0	43	MALE	G3	Stage IV	T2	M1	N0
**TCGA-B0-5120-01A**	493	0	72	FEMALE	G2	Stage I	T1a	M0	N0
**TCGA-B0-5121-01A**	554	0	56	MALE	G2	Stage I	T1b	M0	N0
**TCGA-B0-5399-01A**	652	0	46	MALE	G2	Stage I	T1b	M0	N0
**TCGA-B0-5400-01A**	1132	0	59	FEMALE	G4	Stage III	T3b	M0	N0
**TCGA-B0-5402-01A**	449	0	64	MALE	G4	Stage IV	T4	M0	NX
**TCGA-B0-5690-01A**	2408	0	53	FEMALE	G1	Stage I	T1b	M0	NX
**TCGA-B0-5691-01A**	3431	0	66	FEMALE	G3	Stage I	T1a	M0	N0

**TCGA-B0-5692-01A**	1487	0	66	FEMALE	G3	Stage III	T3b	M0	N0
**TCGA-B0-5694-01A**	480	1	71	MALE	G3	Stage III	T3a	M0	N0
**TCGA-B0-5696-01A**	1727	0	69	MALE	G4	Stage III	T3a	M0	N0
**TCGA-B0-5697-01A**	1835	0	50	MALE	G2	Stage I	T1a	M0	N0
**TCGA-B0-5698-01A**	2583	0	77	MALE	G3	Stage I	T1b	M0	N0
**TCGA-B0-5699-01A**	2741	0	53	MALE	G2	Stage I	T1	M0	N0
**TCGA-B0-5701-01A**	1732	0	65	MALE	G4	Stage III	T3b	M0	N0
**TCGA-B0-5702-01A**	1605	0	71	MALE	G2	Stage I	T1b	M0	N0
**TCGA-B0-5703-01A**	1203	0	73	MALE	G3	Stage I	T1b	M0	N0
**TCGA-B0-5706-01A**	2414	0	45	MALE	G2	Stage II	T2	M0	N0
**TCGA-B0-5707-01A**	2828	0	39	FEMALE	G3	Stage I	T1a	M0	N0
**TCGA-B0-5709-01A**	3117	0	62	FEMALE	G3	Stage III	T3a	M0	NX
**TCGA-B0-5710-01A**	1459	0	57	MALE	G2	Stage I	T1b	M0	N0

**TCGA-B0-5711-01A**	2931	0	50	MALE	G3	Stage III	T3b	M0	NX
**TCGA-B0-5713-01A**	1865	0	75	FEMALE	G3	Stage III	T3b	M0	N0
**TCGA-B2-3924-01A**	371	0	73	MALE	G2	Stage I	T1b	M0	NX
**TCGA-B2-4098-01A**	51	1	72	FEMALE	G2	Stage I	T1b	M0	NX
**TCGA-B2-4099-01A**	374	0	83	MALE	G3	Stage I	T1a	M0	NX
**TCGA-B2-4101-01A**	188	0	52	MALE	G3	Stage II	T2a	M0	NX
**TCGA-B2-4102-01A**	202	0	61	MALE	G2	Stage I	T1b	M0	NX
**TCGA-B2-5633-01A**	358	0	56	MALE	G2	Stage I	T1b	M0	N0
**TCGA-B2-5635-01A**	315	0	74	MALE	G2	Stage I	T1a	M0	NX
**TCGA-B2-5639-01A**	417	0	46	MALE	G3	Stage IV	T3	M1	NX
**TCGA-B2-5641-01A**	324	0	79	MALE	G3	Stage I	T1a	M0	N0
**TCGA-B2-A4SR-01A**	507	0	61	MALE	unknow	Stage II	T2a	M0	NX
**TCGA-B4-5834-01A**	38	0	59	MALE	G1	Stage I	T1	M0	N0

**TCGA-B4-5838-01A**	166	0	52	MALE	G2	unknow	T3	M0	N1
**TCGA-B4-5844-01A**	7	0	61	FEMALE	G1	Stage II	T2	M0	N0
**TCGA-B8-4143-01A**	709	1	66	FEMALE	G3	Stage IV	T3a	M1	N0
**TCGA-B8-4146-01B**	511	0	41	FEMALE	G2	Stage I	T1b	M0	NX
**TCGA-B8-4148-01A**	379	0	63	FEMALE	G3	Stage I	T1a	M0	N0
**TCGA-B8-4154-01A**	255	0	73	FEMALE	G2	Stage I	T1a	M0	N0
**TCGA-B8-4620-01A**	226	0	70	FEMALE	G2	Stage III	T3a	M0	N0
**TCGA-B8-4621-01A**	431	0	63	MALE	G3	Stage I	T1b	M0	N0
**TCGA-B8-4622-01A**	181	0	57	MALE	G3	Stage IV	T3a	M1	N0
**TCGA-B8-5158-01A**	293	0	56	MALE	G4	Stage III	T3a	M0	N1
**TCGA-B8-5159-01A**	240	0	61	FEMALE	G3	Stage I	T1a	M0	N0
**TCGA-B8-5162-01A**	22	0	62	MALE	G2	Stage II	T2a	M0	NX
**TCGA-B8-5163-01A**	4	0	63	FEMALE	G3	Stage III	T3a	M0	N0

**TCGA-B8-5164-01A**	23	0	65	MALE	G3	Stage III	T3a	M0	N0
**TCGA-B8-5165-01A**	8	0	43	MALE	G2	Stage I	T1a	M0	N0
**TCGA-B8-5545-01A**	522	0	42	MALE	G2	Stage I	T1a	M0	N0
**TCGA-B8-5549-01A**	194	0	53	MALE	G3	Stage I	T1b	M0	N0
**TCGA-B8-5550-01A**	434	0	71	MALE	G3	Stage III	T3a	M0	N0
**TCGA-B8-5551-01A**	15	0	65	FEMALE	G3	Stage I	T1b	M0	N0
**TCGA-B8-5552-01B**	392	0	41	FEMALE	G2	Stage I	T1b	M0	NX
**TCGA-B8-5553-01A**	435	0	67	FEMALE	G2	Stage I	T1b	M0	N0
**TCGA-B8-A54D-01A**	830	0	69	MALE	G2	Stage III	T3a	MX	NX
**TCGA-B8-A54F-01A**	519	0	49	FEMALE	G2	Stage I	T1a	MX	NX
**TCGA-B8-A54G-01A**	53	0	50	MALE	G3	Stage I	T1a	MX	NX
**TCGA-B8-A54H-01A**	256	0	69	FEMALE	G3	Stage II	T2a	MX	N0
**TCGA-B8-A54I-01A**	150	0	48	MALE	G3	Stage I	T1b	MX	NX

**TCGA-B8-A8YJ-01A**	431	0	60	FEMALE	G2	Stage I	T1b	unknow	NX
**TCGA-BP-4159-01A**	2601	1	70	MALE	G2	Stage I	T1b	M0	N0
**TCGA-BP-4160-01A**	2881	0	67	MALE	G2	Stage III	T3a	M0	N0
**TCGA-BP-4161-01A**	2746	0	74	MALE	G3	Stage I	T1b	M0	NX
**TCGA-BP-4162-01A**	3074	0	65	FEMALE	G2	Stage I	T1b	M0	N0
**TCGA-BP-4163-01A**	2839	0	60	FEMALE	G3	Stage III	T3a	M0	N0
**TCGA-BP-4165-01A**	3037	0	64	FEMALE	G1	Stage I	T1b	M0	N0
**TCGA-BP-4166-01A**	13	0	69	MALE	G3	Stage III	T3a	M0	N0
**TCGA-BP-4167-01A**	2718	0	59	MALE	G2	Stage III	T3a	M0	NX
**TCGA-BP-4169-01A**	701	1	76	FEMALE	G2	Stage II	T2	M0	N0
**TCGA-BP-4170-01A**	2343	1	72	FEMALE	G2	Stage I	T1b	M0	N0
**TCGA-BP-4173-01A**	1893	0	47	MALE	G3	Stage II	T2	M0	N0
**TCGA-BP-4174-01A**	1879	0	49	MALE	G3	Stage II	T2	M0	N0

**TCGA-BP-4176-01A**	1955	0	64	MALE	G2	Stage I	T1b	M0	NX
**TCGA-BP-4325-01A**	2964	0	64	FEMALE	G2	Stage I	T1b	M0	N0
**TCGA-BP-4326-01A**	1625	1	53	FEMALE	G2	Stage I	T1b	M0	N0
**TCGA-BP-4327-01A**	109	1	75	FEMALE	G2	Stage II	T2	M0	N0
**TCGA-BP-4329-01A**	845	1	75	MALE	G2	Stage III	T3a	M0	N0
**TCGA-BP-4330-01A**	1888	0	60	FEMALE	G2	Stage III	T3a	M0	N0
**TCGA-BP-4331-01A**	2454	1	52	MALE	G2	Stage I	T1a	M0	N0
**TCGA-BP-4332-01A**	1133	0	36	MALE	G2	Stage III	T3a	M0	N0
**TCGA-BP-4335-01A**	475	1	65	FEMALE	G3	Stage IV	T3a	M1	N0
**TCGA-BP-4337-01A**	2	1	76	FEMALE	G4	Stage III	T3b	M0	N0
**TCGA-BP-4338-01A**	2859	0	43	MALE	G3	Stage I	T1b	M0	N0
**TCGA-BP-4342-01A**	2256	1	79	MALE	G3	Stage II	T2	M0	N0
**TCGA-BP-4343-01A**	1912	1	64	MALE	G3	Stage III	T3a	M0	N0

**TCGA-BP-4345-01A**	1516	0	62	MALE	G3	Stage III	T3b	M0	N0
**TCGA-BP-4346-01A**	1493	1	57	MALE	G3	Stage III	T3b	M0	N0
**TCGA-BP-4351-01A**	970	0	51	FEMALE	G2	Stage III	T3a	M0	N0
**TCGA-BP-4353-01A**	375	1	61	MALE	G2	Stage I	T1	M0	N0
**TCGA-BP-4354-01A**	1034	1	40	MALE	G4	Stage IV	T4	M1	N1
**TCGA-BP-4756-01A**	374	0	62	FEMALE	G2	Stage I	T1b	M0	N0
**TCGA-BP-4758-01A**	2208	0	40	MALE	G2	Stage I	T1a	M0	NX
**TCGA-BP-4759-01A**	2372	0	50	MALE	G2	Stage I	T1a	M0	NX
**TCGA-BP-4760-01A**	2361	0	69	MALE	G2	Stage I	T1a	M0	NX
**TCGA-BP-4761-01A**	182	0	57	MALE	G4	Stage III	T3a	M0	N1
**TCGA-BP-4762-01A**	1343	1	42	MALE	G3	Stage I	T1a	M0	NX
**TCGA-BP-4763-01A**	1270	1	79	FEMALE	G2	Stage I	T1a	M0	NX
**TCGA-BP-4766-01A**	1462	0	43	FEMALE	G3	Stage I	T1a	M0	NX

**TCGA-BP-4770-01A**	329	1	73	FEMALE	G4	Stage IV	T4	M0	N0
**TCGA-BP-4771-01A**	162	1	62	MALE	G4	Stage IV	T3a	M1	N0
**TCGA-BP-4776-01A**	411	0	52	MALE	G2	Stage I	T1a	M0	NX
**TCGA-BP-4777-01A**	1731	0	46	MALE	G3	Stage I	T1a	M0	NX
**TCGA-BP-4781-01A**	2080	0	78	MALE	G3	Stage I	T1a	M0	NX
**TCGA-BP-4782-01A**	354	0	55	FEMALE	G2	Stage I	T1a	M0	NX
**TCGA-BP-4787-01A**	480	1	59	FEMALE	G4	Stage IV	T3a	M1	N0
**TCGA-BP-4790-01A**	1111	1	76	MALE	G2	Stage I	T1a	M0	NX
**TCGA-BP-4795-01A**	620	0	74	FEMALE	G2	Stage I	T1a	M0	N0
**TCGA-BP-4797-01A**	1107	0	34	MALE	G3	Stage III	T3b	M0	N0
**TCGA-BP-4798-01A**	334	1	74	MALE	G4	unknow	T3b	M1	N0
**TCGA-BP-4799-01A**	1133	1	70	MALE	G3	Stage III	T3b	M0	N0
**TCGA-BP-4801-01A**	1124	0	57	MALE	G2	Stage I	T1a	M0	NX

**TCGA-BP-4803-01A**	204	0	79	MALE	G3	Stage III	T3a	M0	NX
**TCGA-BP-4804-01A**	1459	0	59	MALE	G2	Stage I	T1b	M0	NX
**TCGA-BP-4807-01A**	211	0	42	MALE	G3	Stage I	T1a	M0	NX
**TCGA-BP-4959-01A**	2660	0	49	MALE	G3	Stage I	T1b	M0	NX
**TCGA-BP-4960-01A**	2172	0	46	MALE	G3	Stage II	T2	M0	N0
**TCGA-BP-4961-01A**	1935	0	47	MALE	G2	Stage I	T1a	M0	NX
**TCGA-BP-4962-01A**	1785	0	58	MALE	G2	Stage II	T2	M0	NX
**TCGA-BP-4963-01A**	1834	0	63	MALE	G3	Stage I	T1b	M0	NX
**TCGA-BP-4964-01A**	1862	0	54	FEMALE	G2	Stage I	T1a	M0	N0
**TCGA-BP-4965-01A**	1871	0	46	MALE	G2	Stage I	T1a	M0	NX
**TCGA-BP-4968-01A**	1746	0	40	MALE	G3	Stage I	T1b	M0	N0
**TCGA-BP-4970-01A**	433	0	44	MALE	G3	Stage III	T1a	M0	N1
**TCGA-BP-4971-01A**	1487	0	40	MALE	G3	Stage III	T3a	M0	N0

**TCGA-BP-4972-01A**	1502	0	43	FEMALE	G3	Stage III	T3a	M0	NX
**TCGA-BP-4973-01A**	1384	0	47	MALE	G3	Stage III	T3a	M0	NX
**TCGA-BP-4975-01A**	1433	0	40	MALE	G3	Stage I	T1b	M0	NX
**TCGA-BP-4977-01A**	454	0	57	MALE	G3	Stage I	T1b	M0	NX
**TCGA-BP-4981-01A**	1097	1	75	FEMALE	G3	Stage III	T3a	M0	NX
**TCGA-BP-4982-01A**	1014	0	42	MALE	G3	Stage I	T1b	M0	NX
**TCGA-BP-4983-01A**	1413	0	67	FEMALE	G4	Stage III	T3a	M0	NX
**TCGA-BP-4985-01A**	952	1	72	MALE	G4	Stage III	T3a	M0	N0
**TCGA-BP-4986-01A**	785	0	75	MALE	G3	Stage I	T1a	M0	N0
**TCGA-BP-4987-01A**	1124	0	41	FEMALE	G2	Stage I	T1b	M0	NX
**TCGA-BP-4989-01A**	118	0	58	MALE	G3	Stage III	T3a	M0	N0
**TCGA-BP-4991-01A**	1413	0	54	MALE	G2	Stage I	T1a	M0	NX
**TCGA-BP-4992-01A**	501	0	66	MALE	G4	Stage I	T1b	M0	NX

**TCGA-BP-4993-01A**	177	0	58	MALE	G3	Stage I	T1a	M0	NX
**TCGA-BP-4995-01A**	1371	0	68	MALE	G3	Stage I	T1b	M0	N0
**TCGA-BP-4998-01A**	932	0	49	MALE	G3	Stage I	T1a	M0	NX
**TCGA-BP-4999-01A**	1266	0	56	MALE	G2	Stage I	T1a	M0	NX
**TCGA-BP-5000-01A**	563	0	40	MALE	G3	Stage I	T1b	M0	NX
**TCGA-BP-5004-01A**	1126	0	53	MALE	G3	Stage I	T1a	M0	NX
**TCGA-BP-5007-01A**	1140	0	45	MALE	G2	Stage II	T2	M0	N0
**TCGA-BP-5008-01A**	1071	0	46	MALE	G2	Stage I	T1a	M0	NX
**TCGA-BP-5009-01A**	1092	1	52	MALE	G3	Stage I	T1b	M0	NX
**TCGA-BP-5010-01A**	878	1	63	MALE	G4	Stage III	T3a	M0	N0
**TCGA-BP-5168-01A**	1463	1	75	MALE	G2	Stage I	T1a	M0	NX
**TCGA-BP-5169-01A**	193	0	70	MALE	G4	Stage I	T1b	M0	N0
**TCGA-BP-5170-01A**	2412	0	55	MALE	G2	Stage I	T1a	M0	NX

**TCGA-BP-5173-01A**	62	1	75	MALE	G2	Stage I	T1a	M0	NX
**TCGA-BP-5175-01A**	932	0	60	MALE	G3	Stage I	T1a	M0	NX
**TCGA-BP-5176-01A**	1590	1	78	FEMALE	G2	Stage I	T1a	M0	NX
**TCGA-BP-5177-01A**	293	0	46	FEMALE	G3	Stage I	T1a	M0	NX
**TCGA-BP-5178-01A**	1912	1	71	MALE	G4	Stage IV	T3a	M1	NX
**TCGA-BP-5180-01A**	2263	0	53	MALE	G2	Stage I	T1a	M0	NX
**TCGA-BP-5181-01A**	1495	0	58	FEMALE	G2	Stage I	T1b	M0	NX
**TCGA-BP-5182-01A**	1165	0	56	MALE	G3	Stage I	T1a	M0	N0
**TCGA-BP-5183-01A**	1291	0	57	MALE	G3	Stage III	T3a	M0	NX
**TCGA-BP-5184-01A**	1133	0	54	MALE	G3	Stage I	T1a	M0	NX
**TCGA-BP-5186-01A**	693	0	50	FEMALE	G2	Stage I	T1a	M0	N0
**TCGA-BP-5187-01A**	406	0	54	MALE	G2	Stage I	T1a	M0	NX
**TCGA-BP-5189-01A**	822	1	60	MALE	G4	Stage I	T1b	M0	NX

**TCGA-BP-5190-01A**	1011	0	61	MALE	G3	Stage I	T1a	M0	NX
**TCGA-BP-5191-01A**	967	0	79	MALE	G2	Stage III	T3a	M0	N0
**TCGA-BP-5195-01A**	749	0	75	MALE	G2	Stage I	T1a	M0	NX
**TCGA-BP-5196-01A**	1018	0	53	MALE	G2	Stage I	T1a	M0	NX
**TCGA-BP-5198-01A**	603	0	72	MALE	G3	Stage III	T3b	M0	N0
**TCGA-BP-5199-01A**	1355	0	58	MALE	G4	Stage II	T2	M0	N0
**TCGA-BP-5200-01A**	1063	0	44	MALE	G4	Stage II	T2	M0	NX
**TCGA-BP-5201-01A**	951	0	63	MALE	G4	Stage IV	T3b	M1	N0
**TCGA-BP-5202-01A**	29	0	75	MALE	G2	Stage III	T3a	M0	NX
**TCGA-CJ-4634-01A**	1820	0	60	FEMALE	G2	Stage I	T1b	M0	NX
**TCGA-CJ-4635-01A**	1416	0	48	MALE	G3	Stage I	T1b	M0	NX
**TCGA-CJ-4636-01A**	1924	0	51	MALE	G3	Stage III	T3a	M0	N0
**TCGA-CJ-4637-01A**	2227	1	52	FEMALE	G4	Stage IV	T2b	M1	NX

**TCGA-CJ-4639-01A**	2308	0	49	FEMALE	G2	Stage II	T2	M0	N0
**TCGA-CJ-4640-01A**	1998	0	49	MALE	G4	Stage III	T3a	M0	N0
**TCGA-CJ-4641-01A**	1661	1	55	FEMALE	G4	Stage IV	T3a	M1	NX
**TCGA-CJ-4642-01B**	1628	0	47	MALE	G2	Stage II	T2	M0	NX
**TCGA-CJ-4643-01A**	1793	0	67	FEMALE	G3	Stage II	T2b	M0	N0
**TCGA-CJ-4644-01A**	336	1	48	FEMALE	G3	Stage IV	T3a	M1	N0
**TCGA-CJ-4868-01A**	646	1	42	MALE	G3	Stage IV	T3a	M1	N0
**TCGA-CJ-4869-01A**	2554	0	49	MALE	G2	Stage III	T2	M0	N1
**TCGA-CJ-4870-01A**	1498	0	58	FEMALE	G2	Stage III	T3a	M0	NX
**TCGA-CJ-4871-01A**	2423	0	63	MALE	G4	Stage IV	T3a	M1	NX
**TCGA-CJ-4872-01A**	1435	0	51	MALE	G4	Stage I	T1b	M0	N0
**TCGA-CJ-4873-01A**	1776	0	85	FEMALE	G3	Stage III	T3a	M0	N0
**TCGA-CJ-4874-01A**	2283	0	73	FEMALE	G3	Stage I	T1b	M0	N0

**TCGA-CJ-4875-01A**	2353	0	67	MALE	G3	Stage IV	T3a	M1	NX
**TCGA-CJ-4876-01A**	1955	0	57	MALE	G3	Stage II	T2b	M0	N0
**TCGA-CJ-4881-01A**	2014	0	41	MALE	G3	Stage III	T3a	M0	NX
**TCGA-CJ-4882-01A**	1883	0	57	MALE	G3	Stage III	T3a	M0	NX
**TCGA-CJ-4884-01A**	1759	0	72	FEMALE	G3	Stage III	T3a	M0	NX
**TCGA-CJ-4886-01A**	1952	0	42	FEMALE	G3	Stage I	T1a	M0	NX
**TCGA-CJ-4887-01A**	932	1	48	MALE	G3	Stage IV	T3a	M1	NX
**TCGA-CJ-4888-01A**	1567	1	59	MALE	G4	Stage IV	T3a	M1	NX
**TCGA-CJ-4889-01A**	1946	0	63	FEMALE	G4	Stage I	T1a	M0	NX
**TCGA-CJ-4890-01A**	2085	0	72	MALE	G4	Stage IV	T3a	M1	N0
**TCGA-CJ-4891-01A**	819	1	57	FEMALE	G4	Stage III	T3c	M0	N0
**TCGA-CJ-4892-01A**	1521	0	65	FEMALE	G2	Stage I	T1b	M0	N0
**TCGA-CJ-4893-01A**	750	0	76	FEMALE	G3	Stage I	T1b	M0	NX

**TCGA-CJ-4894-01A**	841	1	58	MALE	G3	Stage III	T3a	M0	N0
**TCGA-CJ-4895-01A**	1200	1	62	MALE	G4	Stage IV	T3a	M1	NX
**TCGA-CJ-4897-01A**	1808	0	79	FEMALE	G3	Stage III	T3a	M0	NX
**TCGA-CJ-4899-01A**	1528	0	42	MALE	G2	Stage I	T1b	M0	NX
**TCGA-CJ-4900-01A**	1714	1	69	FEMALE	G4	Stage IV	T4	M1	N1
**TCGA-CJ-4901-01A**	1450	0	47	MALE	G3	Stage III	T3b	M0	NX
**TCGA-CJ-4902-01A**	1520	0	61	MALE	G3	Stage III	T3a	M0	NX
**TCGA-CJ-4903-01A**	1560	0	50	MALE	G3	Stage I	T1b	M0	NX
**TCGA-CJ-4904-01A**	1792	0	60	FEMALE	G3	Stage IV	T3a	M1	N0
**TCGA-CJ-4905-01A**	1496	0	62	FEMALE	G2	Stage I	T1a	M0	NX
**TCGA-CJ-4907-01A**	1499	0	58	MALE	G3	Stage III	T3b	M0	NX
**TCGA-CJ-4908-01A**	1531	0	38	MALE	G2	Stage I	T1a	M0	NX
**TCGA-CJ-4912-01A**	1657	0	61	MALE	G3	Stage II	T2	M0	NX

**TCGA-CJ-4916-01A**	1373	0	69	FEMALE	G3	Stage III	T3a	M0	NX
**TCGA-CJ-4918-01A**	93	1	64	MALE	G4	Stage IV	T3a	M1	N0
**TCGA-CJ-4920-01A**	139	1	64	FEMALE	G2	Stage I	T1b	M0	NX
**TCGA-CJ-5671-01A**	1943	0	51	MALE	G3	Stage I	T1a	M0	NX
**TCGA-CJ-5672-01A**	1972	1	84	MALE	G3	Stage I	T1a	M0	NX
**TCGA-CJ-5675-01A**	2430	0	70	MALE	G3	Stage II	T2a	M0	NX
**TCGA-CJ-5676-01A**	2575	0	47	MALE	G3	Stage III	T3b	M0	NX
**TCGA-CJ-5677-01A**	782	1	54	FEMALE	G4	Stage IV	T3a	M1	NX
**TCGA-CJ-5678-01A**	574	1	62	MALE	G3	Stage IV	T2b	M1	N0
**TCGA-CJ-5679-01A**	679	1	73	MALE	G4	Stage III	T3b	M0	NX
**TCGA-CJ-5680-01A**	768	1	65	FEMALE	G4	Stage IV	T3a	M1	NX
**TCGA-CJ-5681-01A**	552	1	44	FEMALE	G3	Stage IV	T3a	M1	NX
**TCGA-CJ-5682-01A**	1883	0	60	MALE	G4	Stage IV	T3a	M1	NX

**TCGA-CJ-5684-01A**	2231	0	61	MALE	G2	Stage III	T3a	M0	NX
**TCGA-CJ-5686-01A**	2038	0	59	FEMALE	G3	Stage I	T1b	M0	NX
**TCGA-CJ-5689-01A**	0	1	90	MALE	G4	Stage I	T1b	M0	NX
**TCGA-CJ-6027-01A**	1855	0	77	MALE	G4	Stage I	T1a	M0	NX
**TCGA-CJ-6028-01A**	1625	1	58	MALE	G4	Stage IV	T3a	M1	NX
**TCGA-CJ-6030-01A**	2299	1	65	MALE	G3	Stage I	T1a	M0	N0
**TCGA-CJ-6031-01A**	1906	0	54	MALE	G3	Stage I	T1b	M0	NX
**TCGA-CJ-6033-01A**	224	1	54	FEMALE	G4	Stage IV	T3a	M1	N0
**TCGA-CW-5580-01A**	1964	1	73	FEMALE	G3	Stage IV	T3a	M1	NX
**TCGA-CW-5581-01A**	2799	0	44	MALE	G3	Stage I	T1b	M0	NX
**TCGA-CW-5584-01A**	164	1	74	MALE	G3	Stage III	T3b	M0	N1
**TCGA-CW-5587-01A**	2226	0	62	FEMALE	G2	Stage III	T3b	M0	N0
**TCGA-CW-5588-01A**	2017	0	78	FEMALE	G2	Stage I	T1a	M0	NX

**TCGA-CW-5589-01A**	2378	0	52	MALE	G2	Stage I	T1a	M0	NX
**TCGA-CW-5590-01A**	1075	1	51	MALE	G3	Stage IV	T3a	M1	NX
**TCGA-CW-6087-01A**	41	1	61	MALE	G4	Stage IV	T3a	M1	N1
**TCGA-CW-6090-01A**	2552	0	68	MALE	G3	Stage I	T1b	M0	NX
**TCGA-CW-6097-01A**	571	1	32	MALE	G4	Stage III	T3a	M0	NX
**TCGA-CZ-4853-01A**	774	0	82	MALE	G2	Stage I	T1a	M0	NX
**TCGA-CZ-4854-01A**	1404	1	68	MALE	G2	Stage I	T1b	M0	N0
**TCGA-CZ-4856-01A**	18	0	62	FEMALE	G2	Stage I	T1b	M0	N0
**TCGA-CZ-4857-01A**	1432	1	56	MALE	G3	Stage IV	T3a	M1	N0
**TCGA-CZ-4858-01A**	1943	0	39	MALE	G4	Stage II	T2	M0	NX
**TCGA-CZ-4860-01A**	206	1	60	MALE	G4	Stage IV	T4	M1	NX
**TCGA-CZ-4861-01A**	446	1	63	MALE	G2	Stage II	T2	M0	NX
**TCGA-CZ-4862-01A**	1843	0	46	MALE	G2	Stage I	T1b	M0	NX

**TCGA-CZ-4863-01A**	1691	0	51	FEMALE	G3	Stage III	T3b	M0	N0
**TCGA-CZ-4864-01A**	1315	1	86	MALE	G3	Stage II	T2	M0	N0
**TCGA-CZ-4865-01A**	166	1	70	FEMALE	G2	Stage I	T1a	M0	NX
**TCGA-CZ-4866-01A**	1768	0	79	FEMALE	G3	Stage I	T1	M0	NX
**TCGA-CZ-5452-01A**	1556	0	69	MALE	G2	Stage II	T2	M0	N0
**TCGA-CZ-5454-01A**	722	1	63	MALE	G2	Stage IV	T2	M1	N0
**TCGA-CZ-5455-01A**	561	1	63	MALE	G4	Stage IV	T3b	M1	NX
**TCGA-CZ-5456-01A**	1558	0	57	MALE	G3	Stage II	T2	M0	N0
**TCGA-CZ-5457-01A**	1547	0	62	MALE	G4	Stage III	T3a	M0	NX
**TCGA-CZ-5458-01A**	1558	0	43	MALE	G3	Stage III	T3a	M0	NX
**TCGA-CZ-5459-01A**	1515	0	63	MALE	G3	Stage III	T3b	M0	NX
**TCGA-CZ-5461-01A**	330	1	52	MALE	G4	Stage IV	T1b	M1	NX
**TCGA-CZ-5462-01A**	311	1	83	MALE	G3	Stage IV	T1b	M1	NX

**TCGA-CZ-5463-01A**	662	0	76	MALE	G2	Stage II	T2	M0	NX
**TCGA-CZ-5464-01A**	1492	0	69	MALE	G2	Stage IV	T3b	M1	NX
**TCGA-CZ-5465-01A**	1377	0	76	FEMALE	G2	Stage III	T3b	M0	NX
**TCGA-CZ-5466-01A**	685	0	67	MALE	G2	Stage III	T3a	M0	NX
**TCGA-CZ-5467-01A**	73	1	86	FEMALE	G4	Stage III	T3a	M0	N0
**TCGA-CZ-5468-01A**	59	1	84	MALE	G4	Stage IV	T3b	M1	NX
**TCGA-CZ-5470-01A**	0	0	72	FEMALE	G3	Stage II	T2	M0	N0
**TCGA-CZ-5984-01A**	1491	0	51	MALE	G3	Stage I	T1b	M0	N0
**TCGA-CZ-5985-01A**	1629	0	58	MALE	G2	Stage II	T2	M0	N0
**TCGA-CZ-5987-01A**	445	1	60	MALE	G2	Stage IV	T3b	M1	NX
**TCGA-CZ-5988-01A**	693	0	38	MALE	G2	Stage I	T1b	M0	N0
**TCGA-CZ-5989-01A**	1599	0	60	MALE	G2	Stage II	T2	M0	N0
**TCGA-DV-5565-01A**	1329	0	59	MALE	G2	Stage I	T1a	M0	NX

**TCGA-DV-5566-01A**	1398	0	67	FEMALE	G2	Stage I	T1a	M0	NX
**TCGA-DV-5568-01A**	370	0	26	MALE	G2	Stage I	T1a	M0	NX
**TCGA-DV-5569-01A**	355	0	29	FEMALE	G2	Stage I	T1a	M0	NX
**TCGA-DV-5573-01A**	1130	0	41	MALE	G2	Stage I	T1a	M0	NX
**TCGA-DV-5574-01A**	723	0	37	MALE	G2	Stage I	T1a	M0	NX
**TCGA-DV-5575-01A**	1006	0	52	FEMALE	G2	Stage I	T1a	M0	NX
**TCGA-DV-A4VX-01A**	1626	1	59	MALE	G4	Stage IV	T3b	MX	N0
**TCGA-EU-5905-01A**	119	0	67	FEMALE	G3	Stage I	T1	M0	NX
**TCGA-EU-5906-01A**	206	0	55	MALE	G2	Stage I	T1b	M0	NX
**TCGA-G6-A5PC-01A**	242	1	54	FEMALE	G4	Stage IV	T1b	M1	N0
**TCGA-G6-A8L6-01A**	313	1	55	MALE	G3	Stage IV	T2a	MX	NX
**TCGA-G6-A8L7-01A**	2133	0	81	FEMALE	G3	Stage I	T1b	MX	N0
**TCGA-GK-A6C7-01A**	61	0	76	FEMALE	unknow	Stage I	T1a	MX	NX

**TCGA-MM-A563-01A**	591	0	41	MALE	G2	unknow	T3	MX	NX
**TCGA-MM-A564-01A**	607	0	68	MALE	G2	Stage II	T2a	MX	NX
**TCGA-MM-A84U-01A**	700	0	58	FEMALE	G2	Stage I	T1a	MX	NX
**TCGA-MW-A4EC-01A**	498	0	72	FEMALE	G2	Stage I	T1a	MX	NX
**GSM904989**	unknow	unknow	77	FEMALE	G2	unknow	T2	MX	N0
**GSM904991**	unknow	unknow	45	MALE	G2	stage II	T2	M0	N0
**GSM904994**	unknow	unknow	48	FEMALE	G2	stage II	T2	M0	N0
**GSM904996**	unknow	unknow	85	FEMALE	G2	stage I	T1	M0	N0
**GSM904999**	unknow	unknow	72	FEMALE	G2	unknow	T1	MX	NX
**GSM905002**	unknow	unknow	51	MALE	G2	unknow	T1	MX	NX
**GSM905007**	unknow	unknow	79	FEMALE	G2	unknow	T1	MX	N0
**GSM905009**	unknow	unknow	unknow	FEMALE	G2	unknow	T1	MX	N0
**GSM905014**	unknow	unknow	60	MALE	G2	unknow	T1	MX	NX
**GSM905017**	unknow	unknow	80	FEMALE	G1	unknow	T1	MX	NX
**GSM905022**	unknow	unknow	70	MALE	G2	unknow	T1	MX	NX
**GSM905026**	unknow	unknow	59	MALE	G4	unknow	T3	M0	NX
**GSM905029**	unknow	unknow	35	MALE	G3	unknow	T3	M0	NX
**GSM905031**	unknow	unknow	56	FEMALE	G3	unknow	T3	M0	NX
**GSM905034**	unknow	unknow	74	MALE	G4	stage III	T2	M0	N1
**GSM905036**	unknow	unknow	82	FEMALE	G3	stage IV	T3	M1	NX
**GSM905038**	unknow	unknow	52	MALE	G4	stage I	T1	M0	N0
**GSM905043**	unknow	unknow	56	MALE	G4	stage III	T3	M0	N1

**GSM905023**	unknow	unknow	67	MALE	G3	stage III	T3	M0	N0
**GSM905024**	unknow	unknow	73	MALE	G4	stage IV	T3	M1	NX
**GSM905027**	unknow	unknow	52	MALE	G4	stage IV	T4	M1	N1
**GSM905032**	unknow	unknow	74	FEMALE	G4	stage IV	T4	M0	NX
**GSM905041**	unknow	unknow	30	MALE	G3	unknow	T3	M0	NX
**GSM905044**	unknow	unknow	56	MALE	G2	stage IV	T1	M1	NX
**GSM1300064**	unknown	unknown	unknown	unknown	unknown	stage I	unknown	unknown	unknown
**GSM1300066**	unknown	unknown	unknown	unknown	unknown	stage I	unknown	unknown	unknown
**GSM1300068**	unknown	unknown	unknown	unknown	unknown	stage I	unknown	unknown	unknown
**GSM1300070**	unknown	unknown	unknown	unknown	unknown	stage I	unknown	unknown	unknown
**GSM1300072**	unknown	unknown	unknown	unknown	unknown	stage I	unknown	unknown	unknown
**GSM1300074**	unknown	unknown	unknown	unknown	unknown	stage I	unknown	unknown	unknown
**GSM1300076**	unknown	unknown	unknown	unknown	unknown	stage I	unknown	unknown	unknown
**GSM1300078**	unknown	unknown	unknown	unknown	unknown	stage I	unknown	unknown	unknown
**GSM1300080**	unknown	unknown	unknown	unknown	unknown	stage I	unknown	unknown	unknown
**GSM1300084**	unknown	unknown	unknown	unknown	unknown	stage I	unknown	unknown	unknown
**GSM1300086**	unknown	unknown	unknown	unknown	unknown	stage I	unknown	unknown	unknown
**GSM1300088**	unknown	unknown	unknown	unknown	unknown	stage I	unknown	unknown	unknown
**GSM1300090**	unknown	unknown	unknown	unknown	unknown	stage I	unknown	unknown	unknown
**GSM1300096**	unknown	unknown	unknown	unknown	unknown	stage I	unknown	unknown	unknown
**GSM1300104**	unknown	unknown	unknown	unknown	unknown	stage I	unknown	unknown	unknown
**GSM1300106**	unknown	unknown	unknown	unknown	unknown	stage I	unknown	unknown	unknown
**GSM1300108**	unknown	unknown	unknown	unknown	unknown	stage I	unknown	unknown	unknown
**GSM1300110**	unknown	unknown	unknown	unknown	unknown	stage II	unknown	unknown	unknown
**GSM1300112**	unknown	unknown	unknown	unknown	unknown	stage II	unknown	unknown	unknown
**GSM1300114**	unknown	unknown	unknown	unknown	unknown	stage II	unknown	unknown	unknown

**GSM1300116**	unknown	unknown	unknown	unknown	unknown	stage II	unknown	unknown	unknown
**GSM1300120**	unknown	unknown	unknown	unknown	unknown	stage II	unknown	unknown	unknown
**GSM1300122**	unknown	unknown	unknown	unknown	unknown	stage II	unknown	unknown	unknown
**GSM1300124**	unknown	unknown	unknown	unknown	unknown	stage II	unknown	unknown	unknown
**GSM1300126**	unknown	unknown	unknown	unknown	unknown	stage II	unknown	unknown	unknown
**GSM1300128**	unknown	unknown	unknown	unknown	unknown	stage II	unknown	unknown	unknown
**GSM1300130**	unknown	unknown	unknown	unknown	unknown	stage II	unknown	unknown	unknown
**GSM1300132**	unknown	unknown	unknown	unknown	unknown	stage II	unknown	unknown	unknown
**GSM1300134**	unknown	unknown	unknown	unknown	unknown	stage II	unknown	unknown	unknown
**GSM1300138**	unknown	unknown	unknown	unknown	unknown	stage II	unknown	unknown	unknown
**GSM1300141**	unknown	unknown	unknown	unknown	unknown	stage II	unknown	unknown	unknown
**GSM1300143**	unknown	unknown	unknown	unknown	unknown	stage II	unknown	unknown	unknown
**GSM1300147**	unknown	unknown	unknown	unknown	unknown	stage II	unknown	unknown	unknown
**GSM1300148**	unknown	unknown	unknown	unknown	unknown	stage III	unknown	unknown	unknown
**GSM1300150**	unknown	unknown	unknown	unknown	unknown	stage III	unknown	unknown	unknown
**GSM1300152**	unknown	unknown	unknown	unknown	unknown	stage III	unknown	unknown	unknown
**GSM1300154**	unknown	unknown	unknown	unknown	unknown	stage III	unknown	unknown	unknown
**GSM1300158**	unknown	unknown	unknown	unknown	unknown	stage III	unknown	unknown	unknown
**GSM1300162**	unknown	unknown	unknown	unknown	unknown	stage III	unknown	unknown	unknown
**GSM1300164**	unknown	unknown	unknown	unknown	unknown	stage III	unknown	unknown	unknown
**GSM1300166**	unknown	unknown	unknown	unknown	unknown	stage III	unknown	unknown	unknown
**GSM1300168**	unknown	unknown	unknown	unknown	unknown	stage III	unknown	unknown	unknown
**GSM1300170**	unknown	unknown	unknown	unknown	unknown	stage III	unknown	unknown	unknown
**GSM1300172**	unknown	unknown	unknown	unknown	unknown	stage III	unknown	unknown	unknown
**GSM1300174**	unknown	unknown	unknown	unknown	unknown	stage III	unknown	unknown	unknown
**GSM1300176**	unknown	unknown	unknown	unknown	unknown	stage IV	unknown	unknown	unknown

**GSM1300178**	unknown	unknown	unknown	unknown	unknown	stage IV	unknown	unknown	unknown
**GSM1300180**	unknown	unknown	unknown	unknown	unknown	stage IV	unknown	unknown	unknown
**GSM1300182**	unknown	unknown	unknown	unknown	unknown	stage IV	unknown	unknown	unknown
**GSM1300184**	unknown	unknown	unknown	unknown	unknown	stage IV	unknown	unknown	unknown
**GSM1300186**	unknown	unknown	unknown	unknown	unknown	stage IV	unknown	unknown	unknown
**GSM1300188**	unknown	unknown	unknown	unknown	unknown	stage IV	unknown	unknown	unknown
**GSM1300191**	unknown	unknown	unknown	unknown	unknown	stage IV	unknown	unknown	unknown
**GSM1300193**	unknown	unknown	unknown	unknown	unknown	stage IV	unknown	unknown	unknown
**GSM1300195**	unknown	unknown	unknown	unknown	unknown	stage IV	unknown	unknown	unknown
**GSM1300197**	unknown	unknown	unknown	unknown	unknown	stage IV	unknown	unknown	unknown
**GSM1300199**	unknown	unknown	unknown	unknown	unknown	stage IV	unknown	unknown	unknown
**GSM1300201**	unknown	unknown	unknown	unknown	unknown	stage IV	unknown	unknown	unknown
**GSM993984**	unknown	unknown	62	male	G4	unknown	unknown	unknown	unknown
**GSM993986**	unknown	unknown	75	female	G1	unknown	unknown	unknown	unknown
**GSM993988**	unknown	unknown	75	male	G1	unknown	unknown	unknown	unknown
**GSM993990**	unknown	unknown	46	male	G2	unknown	unknown	unknown	unknown
**GSM993992**	unknown	unknown	62	male	G1	unknown	unknown	unknown	unknown
**GSM993994**	unknown	unknown	54	male	G2	unknown	unknown	unknown	unknown
**GSM993998**	unknown	unknown	59	female	G3	unknown	unknown	unknown	unknown
**GSM994000**	unknown	unknown	76	female	G1	unknown	unknown	unknown	unknown
**GSM994005**	unknown	unknown	55	female	G2	unknown	unknown	unknown	unknown
**GSM994010**	unknown	unknown	65	female	G1	unknown	unknown	unknown	unknown
**GSM994011**	unknown	unknown	62	male	G1	unknown	unknown	unknown	unknown
**GSM994013**	unknown	unknown	61	male	G2	unknown	unknown	unknown	unknown
**GSM994015**	unknown	unknown	61	male	G1	unknown	unknown	unknown	unknown
**GSM994017**	unknown	unknown	42	male	G2	unknown	unknown	unknown	unknown

**GSM994019**	unknown	unknown	65	female	G3	unknown	unknown	unknown	unknown
**GSM994021**	unknown	unknown	64	female	G2	unknown	unknown	unknown	unknown
**GSM994023**	unknown	unknown	77	female	G2	unknown	unknown	unknown	unknown
**GSM994025**	unknown	unknown	65	male	G3	unknown	unknown	unknown	unknown
**GSM994027**	unknown	unknown	66	female	G4	unknown	unknown	unknown	unknown
**GSM994031**	unknown	unknown	70	male	G1	unknown	unknown	unknown	unknown
**GSM994033**	unknown	unknown	70	female	G2	unknown	unknown	unknown	unknown
**GSM994035**	unknown	unknown	84	male	G1	unknown	unknown	unknown	unknown
**GSM994037**	unknown	unknown	78	female	G2	unknown	unknown	unknown	unknown
**GSM994039**	unknown	unknown	76	male	G3	unknown	unknown	unknown	unknown
**GSM994043**	unknown	unknown	57	male	G3	unknown	unknown	unknown	unknown
**GSM994045**	unknown	unknown	47	male	G2	unknown	unknown	unknown	unknown
**GSM994047**	unknown	unknown	53	male	G1	unknown	unknown	unknown	unknown
**GSM994049**	unknown	unknown	61	male	G2	unknown	unknown	unknown	unknown
**GSM994051**	unknown	unknown	47	male	G3	unknown	unknown	unknown	unknown
**GSM994053**	unknown	unknown	68	male	G2	unknown	unknown	unknown	unknown
**GSM994055**	unknown	unknown	55	female	G1	unknown	unknown	unknown	unknown
**GSM994057**	unknown	unknown	68	female	G2	unknown	unknown	unknown	unknown
**GSM994060**	unknown	unknown	54	male	G2	unknown	unknown	unknown	unknown
**GSM994061**	unknown	unknown	55	male	G3	unknown	unknown	unknown	unknown
**GSM994063**	unknown	unknown	60	female	G1	unknown	unknown	unknown	unknown
**GSM994067**	unknown	unknown	62	mlae	G1	unknown	unknown	unknown	unknown
**GSM994069**	unknown	unknown	59	female	G3	unknown	unknown	unknown	unknown
**GSM994073**	unknown	unknown	78	male	G2	unknown	unknown	unknown	unknown
**GSM994075**	unknown	unknown	60	male	G3	unknown	unknown	unknown	unknown
**GSM994077**	unknown	unknown	66	female	G2	unknown	unknown	unknown	unknown

**GSM994079**	unknown	unknown	66	female	G1	unknown	unknown	unknown	unknown
**GSM994081**	unknown	unknown	54	male	G3	unknown	unknown	unknown	unknown
**GSM994083**	unknown	unknown	79	male	G2	unknown	unknown	unknown	unknown
**GSM994087**	unknown	unknown	75	male	G2	unknown	unknown	unknown	unknown
**GSM994089**	unknown	unknown	70	male	G2	unknown	unknown	unknown	unknown
**GSM994091**	unknown	unknown	56	male	G4	unknown	unknown	unknown	unknown
**GSM994094**	unknown	unknown	70	male	G4	unknown	unknown	unknown	unknown
**GSM994099**	unknown	unknown	69	male	G2	unknown	unknown	unknown	unknown
**GSM994103**	unknown	unknown	70	female	G3	unknown	unknown	unknown	unknown
**GSM994105**	unknown	unknown	67	female	G3	unknown	unknown	unknown	unknown
**GSM994107**	unknown	unknown	72	male	G2	unknown	unknown	unknown	unknown
**GSM994109**	unknown	unknown	63	male	G3	unknown	unknown	unknown	unknown
**GSM994111**	unknown	unknown	67	female	G2	unknown	unknown	unknown	unknown
**GSM994115**	unknown	unknown	69	female	G2	unknown	unknown	unknown	unknown
**GSM994117**	unknown	unknown	76	female	G3	unknown	unknown	unknown	unknown
**GSM994119**	unknown	unknown	51	male	G1	unknown	unknown	unknown	unknown
**GSM994121**	unknown	unknown	55	male	G4	unknown	unknown	unknown	unknown
**GSM994123**	unknown	unknown	75	male	G2	unknown	unknown	unknown	unknown
**GSM994125**	unknown	unknown	52	female	G2	unknown	unknown	unknown	unknown
**GSM994127**	unknown	unknown	54	male	G1	unknown	unknown	unknown	unknown
**GSM994129**	unknown	unknown	61	male	G2	unknown	unknown	unknown	unknown
**GSM994131**	unknown	unknown	61	female	G1	unknown	unknown	unknown	unknown
**GSM994133**	unknown	unknown	67	male	G4	unknown	unknown	unknown	unknown
**GSM994137**	unknown	unknown	52	female	G4	unknown	unknown	unknown	unknown
**GSM994141**	unknown	unknown	63	male	G2	unknown	unknown	unknown	unknown
**GSM994143**	unknown	unknown	62	male	G2	unknown	unknown	unknown	unknown

**GSM994145**	unknown	unknown	57	male	G3	unknown	unknown	unknown	unknown
**GSM994147**	unknown	unknown	78	female	G3	unknown	unknown	unknown	unknown
**GSM994149**	unknown	unknown	58	female	G3	unknown	unknown	unknown	unknown
**GSM994151**	unknown	unknown	77	female	G2	unknown	unknown	unknown	unknown
**GSM994153**	unknown	unknown	70	female	G4	unknown	unknown	unknown	unknown
**GSM994155**	unknown	unknown	76	female	G3	unknown	unknown	unknown	unknown
**GSM994158**	unknown	unknown	48	male	G2	unknown	unknown	unknown	unknown
**GSM994159**	unknown	unknown	64	male	G1	unknown	unknown	unknown	unknown
**GSM994161**	unknown	unknown	54	male	G2	unknown	unknown	unknown	unknown
**GSM994165**	unknown	unknown	64	female	G2	unknown	unknown	unknown	unknown
**GSM994169**	unknown	unknown	65	female	G2	unknown	unknown	unknown	unknown
**GSM994171**	unknown	unknown	67	male	G3	unknown	unknown	unknown	unknown
**GSM994173**	unknown	unknown	46	female	G2	unknown	unknown	unknown	unknown
**GSM994175**	unknown	unknown	67	female	G2	unknown	unknown	unknown	unknown
**GSM994179**	unknown	unknown	59	female	G3	unknown	unknown	unknown	unknown
**GSM994181**	unknown	unknown	78	male	G3	unknown	unknown	unknown	unknown
**GSM994183**	unknown	unknown	62	male	G3	unknown	unknown	unknown	unknown
